# Redistribution of Flexibility in Stabilizing Antibody Fragment Mutants Follows Le Châtelier’s Principle

**DOI:** 10.1371/journal.pone.0092870

**Published:** 2014-03-26

**Authors:** Tong Li, Malgorzata B. Tracka, Shahid Uddin, Jose Casas-Finet, Donald J. Jacobs, Dennis R. Livesay

**Affiliations:** 1 Department of Bioinformatics and Genomics, University of North Carolina at Charlotte, Charlotte, North Carolina, United States of America; 2 Department of Formulation Sciences, MedImmune Ltd., Cambridge, United Kingdom; 3 Analytical Biochemistry Department, MedImmune LLC, Gaithersburg, Maryland, United States of America; 4 Department of Physics and Optical Science, University of North Carolina at Charlotte, Charlotte, North Carolina, United States of America; Oak Ridge National Laboratory, United States of America

## Abstract

Le Châtelier’s principle is the cornerstone of our understanding of chemical equilibria. When a system at equilibrium undergoes a change in concentration or thermodynamic state (i.e., temperature, pressure, etc.), La Châtelier’s principle states that an equilibrium shift will occur to offset the perturbation and a new equilibrium is established. We demonstrate that the effects of stabilizing mutations on the rigidity ⇔ flexibility equilibrium within the native state ensemble manifest themselves through enthalpy-entropy compensation as the protein structure adjusts to restore the global balance between the two. Specifically, we characterize the effects of mutation to single chain fragments of the anti-lymphotoxin-β receptor antibody using a computational Distance Constraint Model. Statistically significant changes in the distribution of both rigidity and flexibility within the molecular structure is typically observed, where the local perturbations often lead to distal shifts in flexibility and rigidity profiles. Nevertheless, the net gain or loss in flexibility of individual mutants can be skewed. Despite all mutants being exclusively stabilizing in this dataset, increased flexibility is slightly more common than increased rigidity. Mechanistically the redistribution of flexibility is largely controlled by changes in the H-bond network. For example, a stabilizing mutation can induce an increase in rigidity locally due to the formation of new H-bonds, and simultaneously break H-bonds elsewhere leading to increased flexibility distant from the mutation site via Le Châtelier. Increased flexibility within the VH β4/β5 loop is a noteworthy illustration of this long-range effect.

## Introduction

The relationship between protein stability and dynamics is complex. Protein structures are highly cross-linked with nearly optimized H-bond networks [1], yet they are decidedly dynamic [2]. This dichotomy makes it very difficult to predict the effects of individual mutations on protein thermodynamics and dynamics [3], [4], [5], [6]. For example, it is common to view mutations that stabilize proteins as also making them more rigid due to improved packing [7], [8]; however, there are important examples of stabilizing mutations that increase dynamics through entropic stabilization [9]. Moreover, the effects of mutations on protein dynamics can propagate through the molecular network, leading to unexpected long-range changes [10], [11], [12], [13], [14]. Other changes that affect protein stability can similarly reveal the complex relationships between rigidity and thermodynamics. For example, reduced pH destabilizes the serine protease inhibitor eglin c, but makes the structure more compact [15], underscoring that rigidity and stability do not always correlate in a naïve way.

Recently, we quantified the complex character of thermodynamic and mechanical response in a comparative study of 14 chemically and structurally diverse point mutations on human C-type lysozyme stability [16] and flexibility [4] relative to the wild type using the Distance Constraint Model (DCM) [17]. We demonstrated that the mutations have frequent, large, and long-ranged effects on protein flexibility. Therein, the mutants were both stabilizing and destabilizing with melting points, *T_m_*, within ±6 K of the wild type. Over 40% of the residues had significant changes in flexibility, and, surprisingly, the average distance between mutant and effected residue was greater than 17 Å. Interestingly, each mutant exhibited changes in rigidity and flexibility along the backbone that typically were distributed in roughly equal proportions, indicating that the residue-specific responses occurred in such a way as to restore the global balance between rigidity and flexibility.

**Figure 1:**
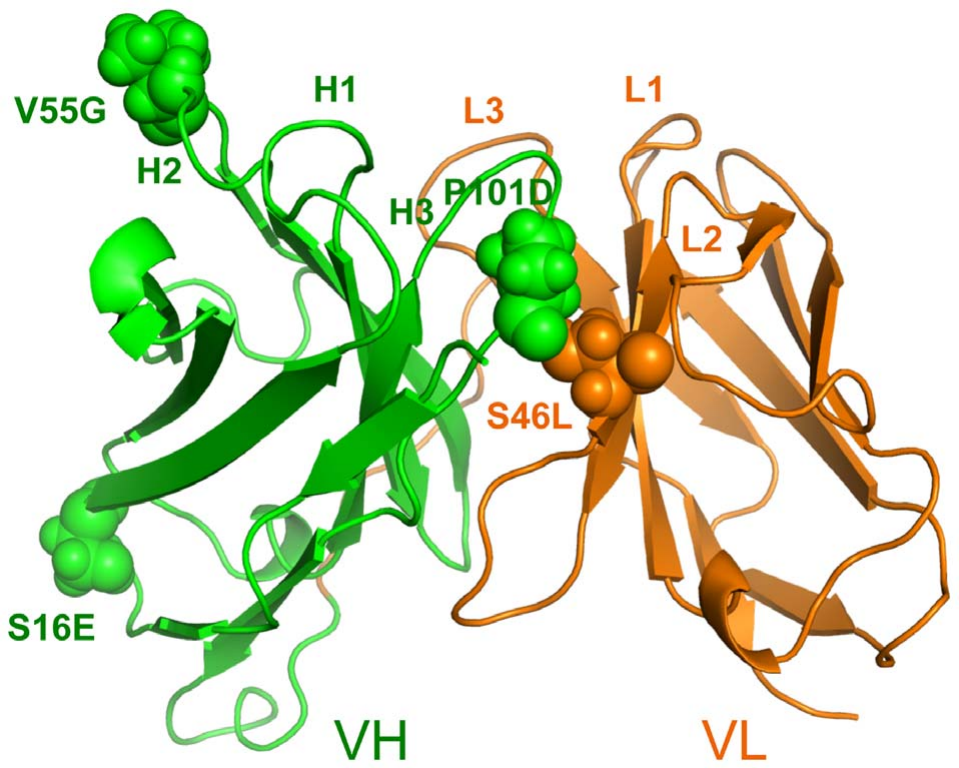
Mutation positions are shown within the anti-lymphotoxin-β receptor (LTβR) antibody single chain Fv fragment (scFv) structure. The wild type structure is shown with VH and VL domains respectively colored green and orange. The complementarity determining regions are labeled and the mutation positions are shown in spacefill with the each mutation labeled adjacently.

In this report, we focus exclusively on the effects of a set of stabilizing mutants within an antibody single chain F_v_ (scFv) fragment system, specifically an anti-lymphotoxin-β receptor (LTβR) antibody [18]. Using an experimental library-screening assay, Miller et al. [19] identified the set by selecting for mutants that increase stability. The five mutants considered here have increases in *T_m_* ranging from 4 to 18 K based on combinations of changes in one to four amino acids (cf. **Table 1**). Importantly, all the mutants were demonstrated to conserve the wild type binding affinity. The mutation locations are shown in **Figure 1**. Similar to our results on lysozyme, we observe a rich mixture of increased rigidity and flexibility along the backbone, and many of these changes are significantly long-ranged. In many instances the mutations lead to local strengthening of the H-bond network. The accompanying loss of conformational entropy due to this increase in rigidity near the mutation site is an enthalpy-entropy compensation mechanism [20] that the DCM captures well through network rigidity [21], [22].

**Table 1:** Dataset statistics.

anti-LTβR scFv mutants	Experimental *T_m_* (K)	Total # of clusters	# conformations in 10 largest clusters
Wild Type	336	18	1802
VH V55G	341	19	1642
VH P101D	345	19	1669
VH S16E; VL S46L	346	19	1906
VH S16E, V55G; VL S46L	351	19	1666
VH S16E, V55G, P101D; VL S46L	354	21	1540

While global rigidification of the native state ensemble can increase thermodynamic stability, it can also be deleterious to function [23]. While not commonly considered, increased flexibility can also entropically stabilize the native state ensemble. However, enthalpy-entropy compensation mechanisms [20] make either extreme improbable. Indeed, across five stabilizing mutant antibody fragments compensating changes in both rigidity and flexibility always occurs because the rigidity ⇔ flexibility equilibria adjust via Le Châtelier to restore the global balance of rigidity and flexibility that is typical within functioning protein structures [24]. This report further establishes enthalpy-entropy compensation frequently occurs far from the mutation site, where weakening the H-bond network in the native state ensemble is compensated by a corresponding increase in flexibility. Our results also indicate that balancing rigidity and flexibility along the backbone is essential for preserving function. These results are consistent the rigidity/flexibility known to occur in a pair of mesophilic/thermophilic RNase orthologs [24], [25], suggesting that this principle has broad applicability.

## Methods

### The Distance Constraint Model

Changes in rigidity are characterized using the DCM, which is based on an all-atom free energy decomposition (FED) scheme combined with constraint theory. Atomic structure is mapped onto constraint topology, where vertices of a graph represent atomic positions and edges describe intramolecular interactions that fix the distance between atomic positions. For a single constraint topology, a Pebble Game (PG) algorithm identifies all rigid and flexible regions [26], [27], which can provide statistically significant explanations of intramolecular couplings [28]. Extending the model, the DCM is based on an ensemble of PG graphs so that fluctuations in constraint topologies due to the breaking and forming of H-bonds and packing interactions are accounted for via a statistical mechanical framework. Specifically, the DCM considers a Gibbs ensemble of PG graphs, each appropriately weighted based on its free energy. The free energy of each graph is calculated from the FED where each constraint is associated with a component enthalpy and entropy. The total enthalpy of a given graph is the sum over the enthalpy contributions from all distance constraints present. However, as described below, the total entropy is calculated in a way that explicitly accounts for nonadditivity [21], [22].

Within the DCM currently applied to proteins [29], [30], the number of native-like torsion constraints, *N_nat_*, and number of H-bond constraints, *N_hb_*, specify a macrostate. Native torsion states have lower energies and entropies relative to disordered torsion states, meaning they correspond to good packing interactions. As a result, protein stability is described in terms of both intramolecular packing and the H-bond network (note: salt bridges are considered to be a special case of H-bonds). The two order parameters, (*N_hb_, N_nat_*), define a macrostate of a protein in terms of its constraint topology, from which a free energy functional is constructed as:
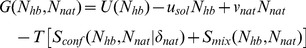
(1)where *U* is the intramolecular H-bond energy, *u_sol_* is an average H-bond energy to solvent that occurs when an intramolecular H-bond breaks, *v_nat_* is the energy associated with a native-like torsion, *S_conf_*(*N_hb_, N_nat_*) is the conformational entropy and *S_mix_*(*N_hb_, N_nat_*) is the mixing entropy of the macrostate associated with the number of ways of distributing *N_nat_* native-torsions and *N_hb_* H-bonds within the constraint topology. There are three phenomenological parameters, {*u_sol_, v_nat_, δ_nat_*}, in the DCM that effectively account for hydrophobic interactions, structural diversity and solvent conditions.

To account for nonadditivity in conformational entropy, *S_conf_* is calculated over the set of independent constraints identified by the PG using:
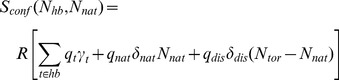
(2)where the index *t* spans over all H-bond constraints in the input structure, and *Rγ_t_* is the entropy contribution when the constraint is independent. Similarly, *Rδ_nat_* and *Rδ_dis_* respectively describe the entropy of a native-like and disordered torsion angle, and *N_tor_* is the total number of torsion angles. The various *q*-values in Eq. (2) represent ensemble averages over constraint topologies for a given macrostate. Within a given constraint topology, each H-bond and torsion constraint has a *q_i_* of either (1 or 0), indicating that the PG identifies the constraint as (independent or redundant). The average *q-*values in Eq. (2) are the conditional probabilities for the constraints to be independent when present. That is, *q-*values are attenuating factors that prevent the DCM from overestimating *S_conf_*. For example, if the PG only identified half of the native torsions as independent across the ensemble of PG graphs, then *q_nat_* = 0.5. In the scFv fragment examples characterized here, the total number of possible PG graphs is greater than 2^1300^, making an exhaustive characterization impossible. As such, Monte Carlo sampling is used to sample networks at each macrostate value (*N_hb_, N_nat_*). Typically, less than 200 samples per macrostate are required for good statistics. Lastly, there is a critically important step that must be executed when determining if a constraint is independent or redundant. When the PG is used to calculate *q_i_* during a recursive process of building the PG graph one constraint at a time [26], the constraints are placed in preferential order from lowest to highest component entropies. With this preferential ordering, the calculation of conformational entropy provides a rigorous lowest possible upper bound. In other words, total conformational entropy reflects the minimal set of the most constrictive yet independent interactions.

Solvation free energy contributions are modeled by the phenomenological *u_sol_* and *v_nat_* parameters [25] that are conjugate to the intramolecular H-bonds and packing order parameters respectively. While mutations are known to quantitatively affect solvation free energies [31], the same *u_sol_* and *v_nat_* parameters are used throughout because the changes are not expected to be large here due to the overall structural similarity across the dataset. Consequently, the solvation free energy differences that *do occur* are reflected in the *T_m_* predictions. We demonstrate below (cf. Results and Discussion) that our *T_m_* predictions are very good, thus indicating that our single parameter set assumption is reasonable.

### Molecular Dynamics Sampling

In prior works, we have used both single [25], [32], [33], [34], [35] and multiple [4] x-ray crystal structural as input to the DCM. The advantage of using multiple structures is that sensitivity to structural artifacts is diminished and uncertainties can be estimated. In this work – for the first time – we employ molecular dynamics (MD) simulations to generate an ensemble of conformations for subsequence DCM analysis. Each mutant and wild type protein is simulated for 100 ns. The MD simulation is done using Gromacs 4.5.5 [36], [37] in the NVT ensemble with the AMBER99SB-ILDN force field [38]. The proteins are solvated by adding 10.0 Å of TIP3P water [39] in a cubic box (counter ions are also added to neutralize charge). Before production, the systems are minimized till convergence or 5,000 iterations, followed by 1 ns of NPT and 1 ns of NVT equilibration. Pressure (1 atm) is regulated using the extended ensemble Parrinello-Rahman approach [40] and temperature (300 K) is controlled by a Nose-Hoover temperature coupling [41], [42]. A nonbonded cutoff of 10.0 Å is used, and Particle-Mesh-Ewald [43] accounts for long-range electrostatic interactions. All bonds to hydrogen atoms in proteins are constrained using LINCS [44], whereas bonds and angles of water molecules are constrained by SETTLE [45], allowing for a time step of 0.002 ps.

**Figure 2:**
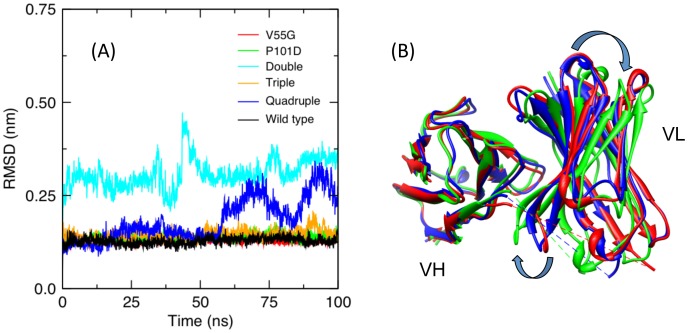
Molecular dynamics trajectories. (A) Root mean square deviations (Cα) are provided for each of the molecular dynamics trajectories. All of the simulations appear well equilibrated, except the quadruple mutant exhibits a continuous change in the orientation between the two domains across the interface. This observation is particularly interesting because the quadruple mutant is the most stable of the five mutants. The slippage along the domain interface is indicated in panel (B), where different colors represent snapshots occurring at: 10 ns (red), 40 ns (blue), and 70 ns (green).

**Figure 3:**
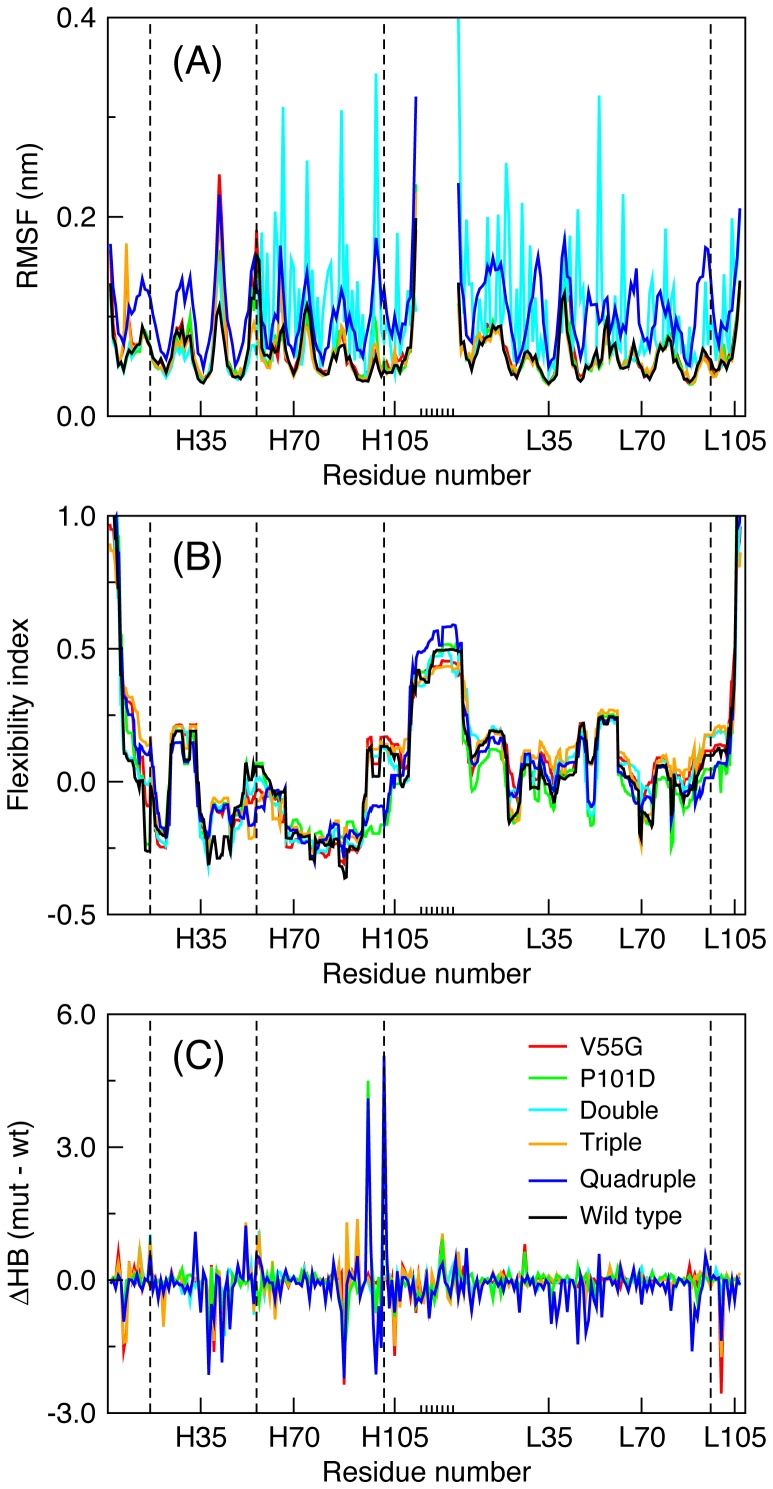
Per residue characteristics. (A) Residue root mean square fluctuations (RMSF) are provided for the six molecular dynamics trajectories. The increased fluctuations within the double and quadruple mutant trivially reflect the VH/VL global rearrangements at the dimer interface. Note that the linker region, indicated by the short tick marks along the x-axis, is ignored because the fluctuations therein obfuscate the rest. The dashed vertical lines indicate where mutations occur. (B) The average flexibility index is provided for each case. Reported values correspond to the appropriate weighted average (defined in methods) over 10 representative structures sampled from the MD trajectory. Changes in flexibility relative to the wild type are both structurally local (as observed at the two highlighted mutant locations) and remote from the mutation site. (C) Differences in H-bond between wild type and each mutant. The total H-bond counts (donor and acceptor) are averaged across the MD simulation for each residue. Note that there is no wild type (black) series in panel (C) because the reported values are differences.

**Figure 2A** plots the root mean square distances (RMSD) for all six MD trajectories. In all but two cases, the trajectories appear perfectly stable. The exceptions are the double (VH S16E; VL S46L) and quadruple (VH S16E, V55G, P101D; VL S46L) mutants. Curiously, despite the increased mobility, the latter has the highest *T_m_* across the dataset. In both cases, the increased conformational rearrangements are due to ‘slippage’ along the domain interface. That is, the relative orientations of the VH and VL domains are continually rearranging (**Figure 2B**), which inflates the per residue root mean square fluctuations (RMSF) for these two examples (cf. **Figure 3A**). Conversely, the fluctuations within the constituent VH and VL domains are relatively minor compared to the global rearrangements (cf. **Figure S1**). Note that the double mutant has one residue change at the domain interface, whereas the quadruple mutant has two, presumably contributing to the conformational frustration. While several studies support the idea that the orientation between the VH and VL domains can affect antigen-binding properties [46], [47], [48], the binding activity of the quadruple mutant is conserved, indicating a functional tolerance for such conformational diversity.

A total of 2,000 evenly spaced frames from each trajectory are clustered using the KCLUST module [49] from the MMTSB tool set [50] based on the RMSD of all CA and CB atoms, except those in the linker. Excluding the highly mobile linker focuses our analysis on core structural rearrangements. **Figure S2** and **Table 1** summarize the number of conformations represented by each cluster. We adjust the cluster radii to maintain around 20 total clusters, where the ten largest represent 77 to 95% of the total conformations. A representative structure is identified as the structure closest to the centroid from each of these ten largest clusters. Each of the ten representative structures is then subsequently energy minimized and used as DCM input. A weighted average of all DCM properties is taken over the ten representative structures, where the total number of structures within the cluster containing a given representative structure defines its weight.

### Quantitative Stability/Flexibility Relationships

In addition to thermodynamic response, the DCM calculates a number of mechanical properties for each framework that describe Quantitative Stability/Flexibility Relationships (QSFR) of the protein. The Boltzmann weights from the partition function adjust with temperature, leading to appropriate thermodynamic averaging of the mechanical quantities. For example, folded and rigid structures, punctuated by flexible loops, are prevalent at low temperatures, whereas the protein is primarily flexible in the denatured ensemble at temperatures greater than the melting temperature, *T_m_*, defined by the heat capacity peak. The backbone Flexibility Index (FI) and the Cooperativity Correlation (CC) serve as useful QSFR metrics for characterizing mechanical properties within a protein [51].

The FI indicates whether a rotatable bond is flexible – because it can rotate as a mechanical *hinge*, or rigid – because it is *locked* due to network constraints. FI is calculated by ensemble averaging over the quantity *f_i_* = (*h_i_* – *l_i_*) defined based on a single constraint topology as follows. When the *i*-th rotatable bond can rotate within a flexible region, the number of rotatable bonds that can rotate (distinct hinge motions) within that flexible region is counted, and denoted as H. The number of independent disordered torsions within that flexible region is also counted, and denoted as A. The value *h_i_* = A/H represents the density of independent DOF within that flexible region, and it is assigned to all H rotatable bonds within. Conversely, if the *i*-th rotatable bond is locked within an over-constrained region, the total number of rotatable bonds that are locked are counted and denoted as L. The number of redundant constraints within that over-constrained region is also counted, and denoted as B. The value *l_i_* = B/L represents the density of redundant constraints within that over-constrained region, and it is assigned to all L locked bonds within. In the special case that B = 0, the locked bond is called isostatic, but this distinction is lost in FI due to ensemble averaging. Note that all constraint networks are treated with equal weight within a given macrostate, but because Boltzmann factors weight macrostates differently within the free energy landscape, the most probable constraint networks depend on temperature. In this report, we average over the macrostates corresponding to the native basin to focus on equilibrium fluctuations in the folded protein at *T = T_m_*.

The CC matrix is calculated similarly to FI; however, mechanical couplings are being tracked. That is, for a given constraint topology, the decomposition of regions as described above also yield which pair of rotatable bonds are in the same flexible region or same rigid region. If the *i*-th and *j*-th rotatable bond are in the same flexible region, the matrix element CC*_ij_* = *h_i_* (recall *h_i_* = *h_j_*). If they are in the same rigid region, the matrix element CC*_ij_* = – *l_i_* (recall *l_i_* = *l_j_*). If the pair of rotatable bonds are not within the same distinct region, the matrix element CC*_ij_* = 0 and this pair of rotatable bonds are not correlated. The size of the CC matrix representing the backbone is nominally 2N×2N because both φ and ψ torsions are tracked along the backbone, but generally it is smaller because proline only has one rotatable bond. Note that the backbone rotatable bonds within a residue can be averaged to arrive at an N×N matrix, but the CC matrix that we typically use, which is the case herein, show all rotatable angles. As a final note, the CC matrix derives from an ensemble average over constraint networks in the same way FI is averaged. Thus, we will focus on CC in the native state of the protein at *T = T_m_*.

### Structure Preparation and Model Parameterization

The initial anti-LTβR scFv structures are modeled from the wild type Fab structure (PDB ID: 3HC0). The Fv and Fc fragments are severed, and the VH and VL domains are joined via a (Gly_4_Ser)_3_ linker using SWISS-MODEL [52]. The side chain prediction program SCWRL4 [53] is used to model the side chains of the mutants. After MD simulation and clustering, hydrogen atoms are added back to each representative structure using H++ [54] to ensure proper ionization by considering residue p*K_a_* values followed by a final minimization.

The phenomenological parameters, {*u_sol_, v_nat_, δ_nat_*}, are obtained by fitting to experimental heat capacity curves from DSC. In a recent report [55], we established parameter ranges for various antibody fragment sizes. Similar to prior work [16], [35], we fit the representative structure corresponding to the largest cluster of the VH P101D mutant to its *C_p_* curve [19]. The model fits the experimental curve very well (cf. **Figure S3**), which gives parameter values of {*u_sol_ = *−2.71 kcal/mol, *v_nat_ = *−0.89 kcal/mol, *δ_nat_ = *1.89}. The same model parameters are then applied to all representative structures for the wild type protein and for all mutants.

### Comparative Analyses

Note that in this report, residue numbering is based on the Kabat scheme [56]. The QSFR properties are calculated for each representative structure, and a second average over the 10 representative structures is performed with a weighting that is based on cluster size. To compare mutant QSFR properties to the wild type, we use a Z-score (Eq. 3) to discern differences between the wild type (w) and mutant (m) results across the 10 representative structures.
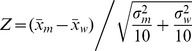
(3)


The averages (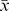) and standard deviations (σ) are obtained using the same cluster weightings. The value of 10 corresponds to the number of representative structures considered. Using a very conservative Z-score cut-off, changes in rigidity are deemed to occur when |Z-scores| are greater than 2.33, corresponding to a p-value of 0.01. Further, large changes are deemed to occur when |Z-scores| are greater than 3.33, which corresponds to a p-value of 0.0005. That is, the odds of a moderate change occurring by random chance are 1 in 100, and the odds of a large change occurring by random chance are less than 1 in 2300. No change is assigned when Z-scores are between ±2.33. It is important to note, that compared to the Z-score thresholds of ±1 used in our lysozyme work where a Z-score [4], these cut-offs are much more conservative.

## Results and Discussion

### Melting Point Prediction

Previously we demonstrated that the DCM is able to reproduce experimental *T_m_* values with an average error of 4.3% (Pearson correlation = 0.64) across a dataset of 14 lysozyme point mutants [16] based on a single parameterization using experimental heat capacity data from any mutant or the wild type. Due to the phenomenological nature of the model, we also have shown that the parameters *u_sol_* and *v_nat_* are linearly correlated in their variation across proteins. The range for the {*u_sol_*, *v_nat_*} parameters has marked consistency that holds up across diverse proteins that span SCOP class and size variations, including hierarchical complexes of antibody fragments [55]. Encouraged by the overall consistency in DCM parameters, and especially when limited to similar proteins, we again apply a single parameter set across all structures considered.

In this work, we apply the DCM to 10 representative structures generated by molecular dynamics. As such, perturbations within the {*u_sol_*, *v_nat_*} parameters must take place to account for solvation free energy changes as the protein conformation changes. Nevertheless, we maintain fixed parameters for all conformations and all mutants and the wild type. The fixed parameters enforce model consistency across the sampling, so that errors related to solvation effects caused by not perturbing *u_sol_* and *v_nat_* are reflected in the predicted *T_m_* values. Finally, there is uncertainty in the experimental *T_m_* values themselves. This is because three of the scFv proteins (wild type, double and quadruple mutants) thermally unfold with two transitions corresponding to the VH and VL domains [19]; however, in each case the DCM predicts a single transition. For these three proteins, a single *T_m_* was defined as the target by averaging the two *T_m_* values reported. Moreover, while some of the experimental data is available for the scFv fragments, others are only available for the Fv fragment without the linker, which is what is used here. With these caveats in mind, the DCM predicts the *T_m_* values with an average error of 1.1% and a Pearson correlation of 0.72 (cf. **Figure S4**) corresponding to an error of ∼4 K. As such, we are obtaining good accuracy from the DCM despite its mean field description of solvation effects, where molecular details of solvation are combined into two parameters. Interestingly, the error in *T_m_* is greatest in the wild type protein (cf. **Figure S5**), where the DCM predicts it to be too stable.

### Sensitivity of Results on the Number of Representative Structures

**Figure 4:**
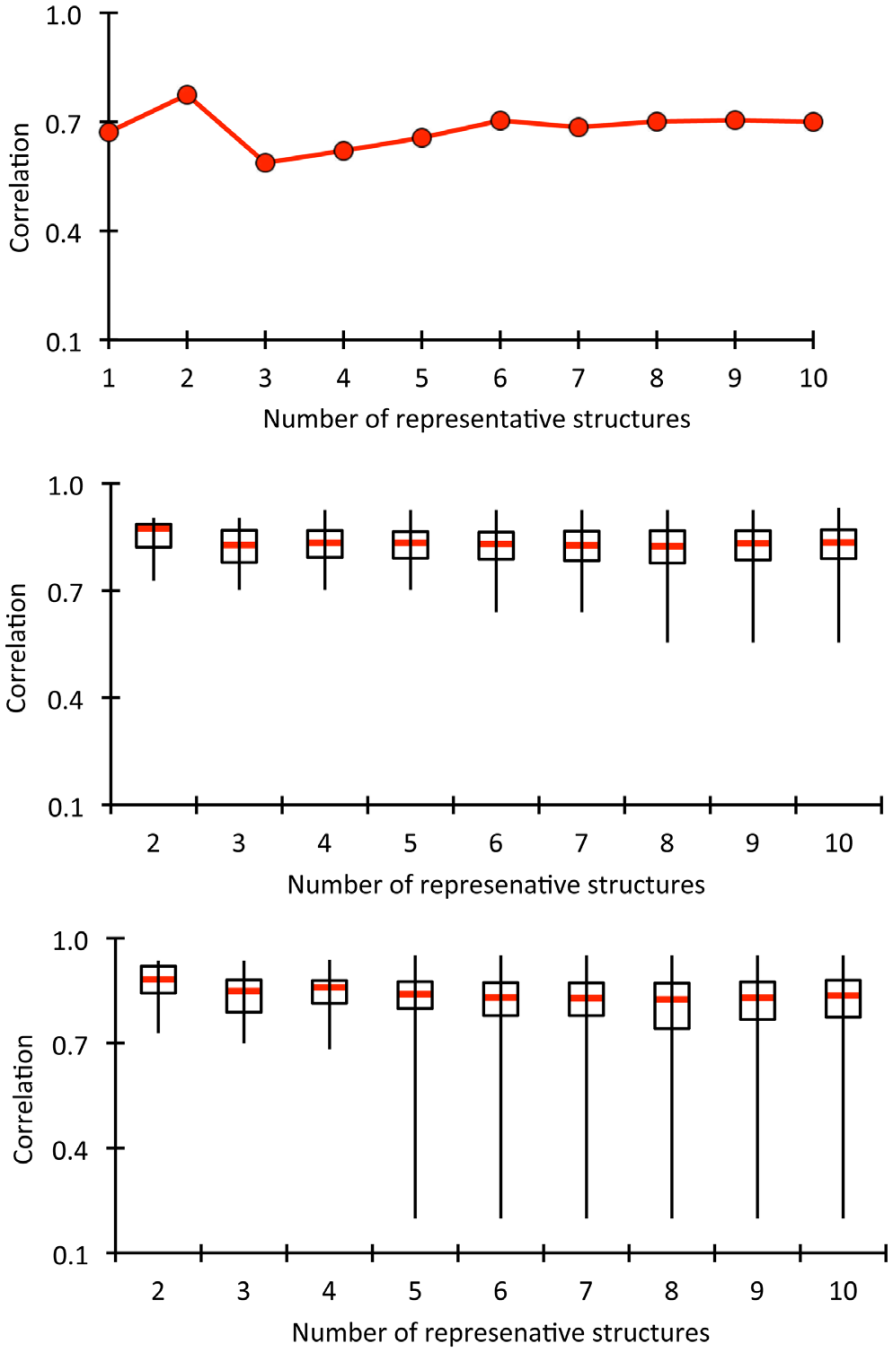
The effects of the number of representative structures on the QSFR results are considered. In panel (A), the accuracy of the *T_m_* predictions are plotted versus number of representative structures used, where accuracy is described the Pearson correlation between the predicted and experimental sets. In panels (B) and (C), the similarity between the backbone flexibility index and cooperativity correlation plots, respectively, are compared for each pair of representative structures with the same sequence. These values are collapsed across the six proteins and their distributions are compared using box plots. The medians and first/third quartiles are very consistent, indicating that mechanical predictions are robust.

The *T_m_* results indicate that the employed hybrid has utility. However a natural question arises about the sensitivity on the number of representative structures (*N_rep_*) used to calculate the weighted average QSFR properties, including *T_m_*. Uncertainties are greatest when only a few representative structures are used. Recall that as *N_rep_* increases the effect of subsequent representative structures successively becomes less as the weight decreases following the rank ordered decreasing cluster sizes. Therefore, **Figure 4A** plots the weighted average *T_m_* prediction as a function of *N_rep_*, which shows *N_rep_*≥6 is sufficient to obtain converged results that agree markedly well with experimental values.

The sensitivity in the predicted FI and CC properties as a function of *N_rep_* is benchmarked. However, unlike with the *T_m_* values, we do not have an experimental standard to compare in these cases. As such, consistency is monitored in the FI and CC values across all possible pairs of representative structures as *N_rep_* goes from 2 to 10. Note that this assessment treats all representative structures equally, whereas the actual averaging approach used by the hybrid method is averaged based on occupancy. Nevertheless, the pairwise comparisons provide a straightforward assessment of the QSFR variability within the representative structure sets, corresponding to an upper bound on the probabilistic variability. **Figure 4B–C** plots the distributions of pairwise Pearson correlations between all pairs of structures for the various *N_rep_* values. While low similarities do occur as rare events for larger values of *N_rep_*, the medians (±1 quartile) of the distributions are very robust, especially for *N_rep_*≥3. Taken together, the above results demonstrate that *T_m_* is robust at *N_rep_*≥6, whereas the mechanical properties are robust at much smaller values of *N_rep_*, which is consistent with our earlier results [25]. Upon careful inspection, low similarity pairs are more frequent in CC, relative to FI, which is also consistent with our prior results [25], [33], [34], [35], [57], [58], [59]. Good convergence to consistent results is obtained when representative structures are selected from each cluster at random (data not shown). Moreover, insensitivity to rigidity properties over an ensemble of PG graphs has been recently verified independently, and exploited through an empirical protein independent parameterization [60] used to characterize protein flexibility. Taken together, these results indicate that an average over the most weighted ten representative structures reduces the statistical variance in mean QSFR properties to a point that is far less than the level of accuracy that can be expected from the employed phenomenological DCM underlying the calculations.

It is important to stress that the primary goal of this work is not to predict *T_m_* values, which are sensitive to the relative probabilities of the native and unfolded basins. Rather, we want to characterize the mechanical response within the native basin across the set of proteins. However, while the DCM allows thermodynamic and mechanical response to be directly linked, solvent penetration as it relates to changes in the H-bond network (HBN) is not modeled in molecular detail. Therefore, we mitigate these concerns by using representative structures from the all-atom MD simulation in explicit solvent, which gives ample opportunity to capture the formation and breaking of intramolecular H-bonds as solvent interacts with the protein. Holding the DCM parameters constant across all proteins and their representative structures provides a means to assess sensitivity in thermodynamic stability as fine details in structure fluctuate at the all-atom level. Indeed, the marked consistency in *T_m_* predictions described above strongly suggests that the change in mechanical response upon mutation can be ascribed to equilibrium shifts within the native state influenced primarily through conformational entropy compensation.

### Rigidity Changes in Anti-LTβR Mutants

Comparisons of mesophilic and thermophilic orthologs pairs have been used extensively to reveal how stability and flexibility are related [61], [62]. While increased rigidity in thermophilic proteins has been reported [7], [8], there are instances where no correlation or inverse correlation is found [63]. The latter case demonstrates increased stability of the native state can be achieved with an increase of conformational entropy by increasing flexibility. Presumably, functional constraints require there to be a balance between stability and flexibility at relative temperatures (e.g., organismal optimal growth temperatures) [24], [25] to conserve critical catalytic mechanisms. Compared to mesophilic/thermophilic orthologs pairs, where the sequence identity typically ranges between 40 and 80%, the stabilizing mutant proteins in our dataset differ by only one to four residues. As a consequence, the complexity of the problem is reduced because a separation between local and spatially long-range effects relative to the point mutation sites is possible.

The MD results reveal the most stable quadruple mutant is very mobile (**Figure 2B**). To understand why, the FI for the wild type and mutant scFv structures are shown in **Figure 3B**. While it could be expected that FI is well conserved, there are interesting and significant differences. Many of the differences are local to the mutation. For example, the VH P101D mutation is present as a single mutant and is also part of the quadruple mutant, causing significant local rigidity changes relative to the four structures without it. There are also widespread nonlocal changes that primarily modulate intensity without necessarily causing a switch from rigid to flexible (or vice versa). This is perhaps best exemplified by the flexibility within the linker region. However, there are a small number of cases with wholesale differences (e.g. serine L50 in CDR L2).

To better assign significance to the observed changes, we recast the differences between the wild type and each mutant as Z-scores. The Z-scores for each mutant are plotted against residue number in **Figure S6** where all rigidity/flexibility differences are classified as no significant change (|Z-score| <2.33), moderate change (2.33< |Z-score| <3.33) and large change (|Z-score|>3.33). **Table S1** counts the number of residues with altered rigidity. Across the dataset the overall number of residues with increased rigidity (42%) is similar to increased flexibility (58%). These percentages show that the expectation that stabilizing mutations rigidify a protein is naïve. Rather, the naïve expectation is but one possible route of stabilization. Interestingly, each mutant is skewed in one direction or the other, where the average in the absolute value of the percent difference between increased rigidity and flexibility is 35%. The triple mutant (VH S16E, V55G; VL S46L) has the greatest skew, where 82% of the changes are increased flexibility.

**Figure 5:**
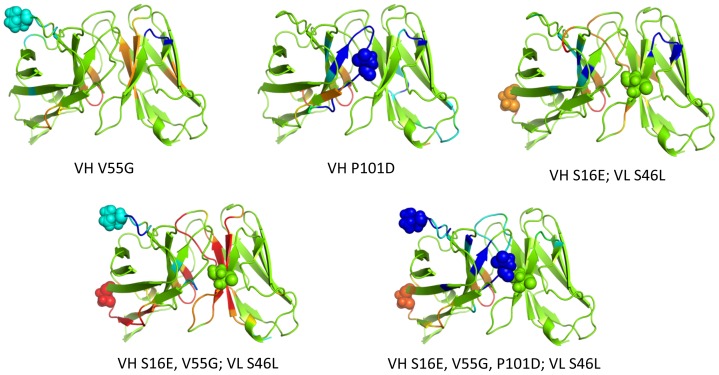
Changes in rigidity within the mutant structures relative to wild type are indicated by color: green = no change; cyan and blue = moderate and large rigidity increases; and orange and red = moderate and large increases in flexibility. In each case, the color represents a certain z-score range for differences that are defined within **Figure S6**.

**Figure 6:**
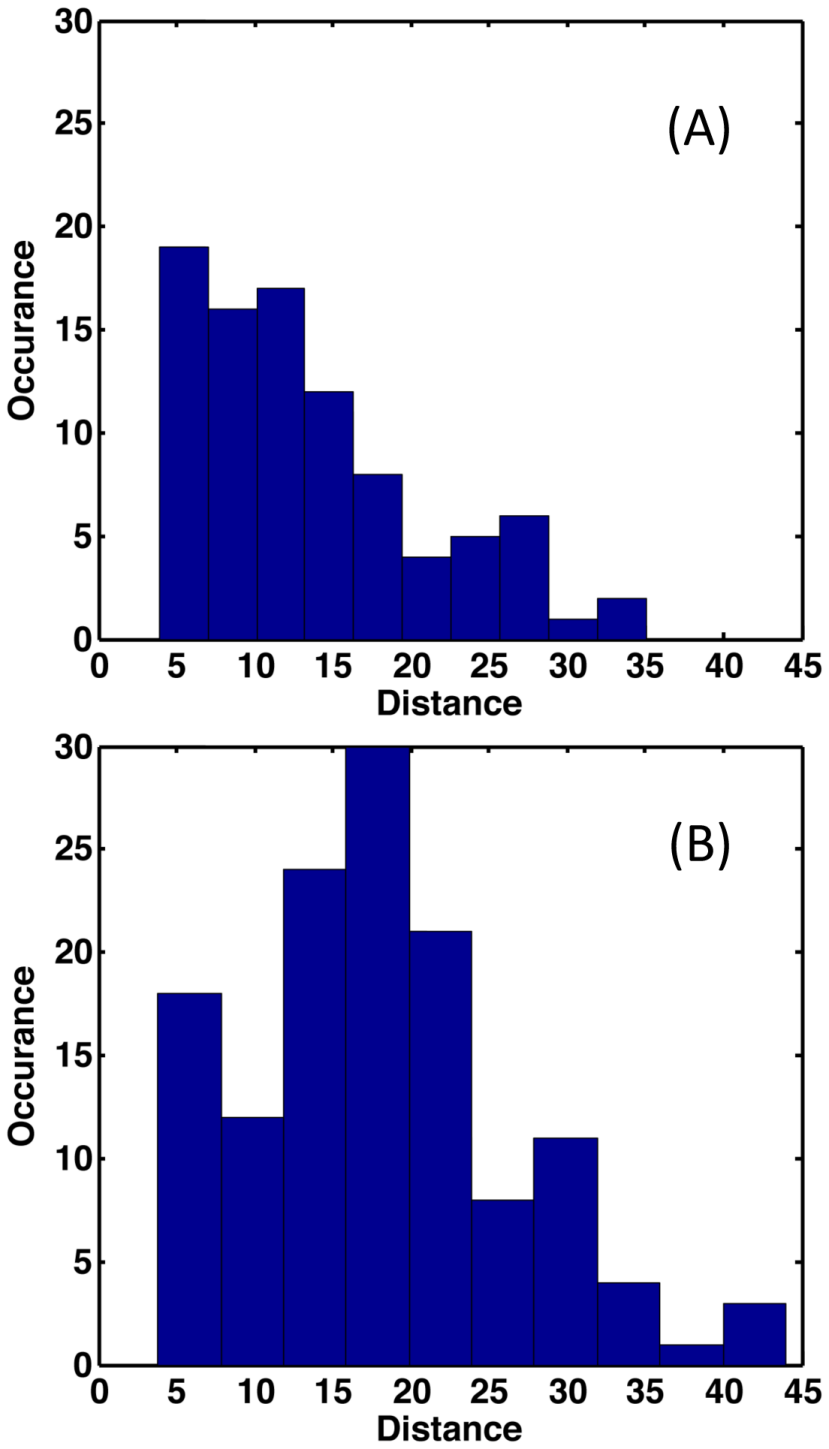
Statistically significant changes in rigidity (p<0.01) are tabulated based on the distance between them and the closest constituent mutation. Note that the average distance in the increased rigidity response (A) is significantly less than increased flexibility (B), 13.6 Å vs. 17.9 Å, respectively.

Mechanical response is conveyed in **Figure 5**, which maps the Z-score classification of rigidity changes on ribbon renderings of the protein structures. Moderate and large increases in rigidity are respectively colored cyan and blue corresponding to p-values of 0.01 and 0.0005; conversely, moderate and large increases in flexibility are respectively colored orange and red (corresponding to the same p-values). Green indicates no statistically significant change is rigidity/flexibility. Note that changes tend to occur primarily in loop regions. Consider the VH V55G mutant, which is in the H2 complementarity-determining region (CDR). While there is some local rigidification of H2, most of the rigidity changes occurring are far removed from the mutation that propagate into the VL domain. Moreover, the response to VH V55G includes a nearly equal mix of both increased rigidity and flexibility. Conversely, the VH P101D mutant primarily rigidifies the protein, whereas the triple mutant primarily increases flexibility, punctuated by relatively few local rigidity increases. In addition to being more slightly more frequent, increases in flexibility are more likely to occur far from the mutation site. **Figure 6** plots histograms summarizing the distances at which the mechanical responses occur. While the vast majority of increases in rigidity occur within a local neighborhood to the mutation site (the average distance from the closest mutation to a residue with increased rigidity is 13.6 Å), the distribution is noticeably shifted to longer distances for increased flexibility (average distance is 17.9 Å).

### H-Bond Network Differences Affect Rigidity

Hydrogen bonds play an important role in protein stability because they stabilize secondary structure and provide critical cross-links that organize the tertiary structure [64]. Since the HBN is a critical component to constraint topology and fluctuations therein, characterizing the HBN changes in response to mutation is critical to understand the observed rigidity differences. Differences in the HBN were previously analyzed in terms of total number present, total energy and corresponding regions between structures to identify where H-bonds break and reform [33], [35]. We also compare densities of H-bonds present along the backbone by invoking the MD trajectories to track H-bonds flickering. HBNs for the wild type and five mutant structures are provided in **Figure S7**.

The HBN differences are greater outside of secondary structures, while secondary structure H-bonds are more robust. This suggests that the preservation of secondary structure H-bonds are largely responsible for backbone flexibility to be well conserved, and why FI aligns well with secondary structure elements. Conversely, the largest H-bond differences involving side chains elucidate significant differences in rigidity properties. That is, a change in a handful of critically placed side chain H-bonds can drastically alter mechanical linkage properties. Some of these differences are visually apparent when comparing the wild type structure to the mutants. For example, the region surrounded by the red circles in **Figure S7** show changes in H-bond densities in the region of the VH P101D mutants. These changes directly lead to local increases in rigidity. More interestingly, owing to their ability to span long stretches of sequence, long-range changes in flexibility should be expected.

While increases in rigidity and flexibility are nearly equal globally, one of the most important observations from our results is that within this dataset increased rigidity is significantly more common than increased flexibility within the CDRs. That is, when there is a response within a CDR, increased rigidity occurs 90% of the time. In stark contrast, the converse is true for non-CDR loops, where increased flexibility is the predominant response (80%). Increased rigidity occurs within CDR H2 in all three mutants with the VH V55G mutation. Similarly, the VH P101D mutation parallels the increased rigidity within H3. In contrast, several non-CDR loops on the distal face of the antibody tend to become more flexible upon mutation.

**Figure 7:**
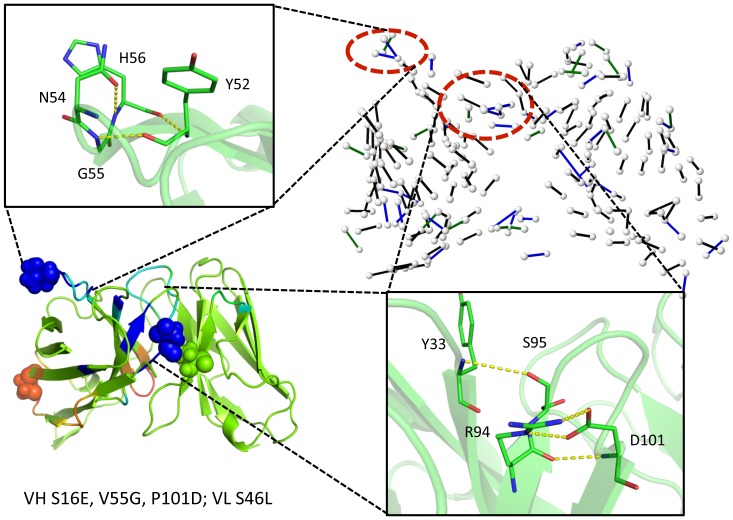
Regions that exhibit large increases in rigidity within the quadruple mutant are identified. The effects of the quadruple mutation (VH S16E, V55G, P101D; VL S46L) on protein flexibility are displayed in the lower left panel. The hydrogen bond network (HBN) is displayed in the upper right panel. Color indicates H-bond frequency across the full MD trajectory, black>90% occupancy, 90%≥blue>70%, and 70%≥green>50% (H-bonds with less than 50% occupancy are not shown). The two red circles emphasize two regions in the HBN with increased number of hydrogen bonds relative to the wild type, leading to an increase in rigidity. That is, the new hydrogen bonds highlighted in yellow dashed lines locally rigidify the corresponding regions, which correspond to complementary determining regions H2 and H3.

Using the quadruple mutant as an example, **Figure 7** clearly demonstrates how the constituent VH V55G and VH P101D mutants lead to increased rigidity within CDRs H2 and H3, respectively. The H-bond network for the mutant structure is shown in the upper-right, and the indicated regions correspond to H-bond density increases therein, relative to wild type, thus rigidifying those regions. Within the H-bond network, color indicates how frequent the H-bond is observed across the full MD trajectory, black>90% occupancy, 90%≥blue>70%, and 70%≥green>50% (H-bonds with less than 50% occupancy are not shown). Mutations tend to locally optimize HBNs, causing a more tightly connected structure. As indicated, there are four new high probability H-bonds in H3 and three new H-bonds in H2. The increased rigidity in CDR H3 is due to the VH P101D change. This response is particularly noteworthy because one might naively assume that loss of the cyclic proline would cause an increase in flexibility. However, in this example, the strain imposed by proline prevents two of the new H-bonds from forming, while two others arise from new interactions with the introduced carboxylate group. The sharp peaks at H98 and H101 in **Figure 3C** correspond to these new H-bonds. A similar response in H3 occurs in the P101D single mutant. Regarding the increased rigidity in CDR H2, Jordan et al. [18] argues that the VH V55G mutation removes unfavorable φ and ψ torsion angle strain within the loop. In agreement, we observe pronounced structural rearrangements in that loop that are stabilized by the new H-bonds.

On the polar opposite end of the scFv fragment, two non-CDR loops in the same structure show significant flexibility increases. First, the VH β2/β3 loop highlighted in yellow in **Figure S8** (residues H12–H17) that includes the VH S16E mutation, leads to the loss of a strong H-bond between serine H16 and lysine H13. Likewise, the VH β4/β5 loop (residues H39–H45) becomes significantly more flexible. Again, HBN comparisons reveal the origins of the rigidity changes (cf. **Figure S9**). Specifically, the increased flexibility is strongly associated with the loss of H-bonds, where a H-bond between glutamine H43 and alanine H40 and a bifurcated H-bond between glutamate H46 and arginine H38 are lost (cf. **Figure 3C**). The observed flexibility increases in the β4/β5 loop are particularly interesting for two reasons. First, increased flexibility occurs in all five mutants, representing the only strictly conserved response across the dataset. Second, these changes cannot simply be ascribed to local events. That is, the observed changes must reflect long-range changes propagating through the molecular networks because none of the mutants occur in this region.

### Changes in Mechanical Couplings

The CC plots characterize mechanical couplings between residue pairs, providing a snapshot of allostery. It is worth pointing out that a particular rigid cluster can itself be very mobile, indicating the motion of residues therein are highly correlated through the rigid body movement. When a pair of residues is flexibly correlated, random thermal motions of one residue are readily channeled into pathways dictated by how flexibility propagates through the protein to affect conformational change of the other residue, and vice versa. The rigidity network analysis highlights pathways defined by the native state ensemble of constraint topologies, but the mobility of atoms is not determined. Note, however, that molecular contacts can decrease mobility within flexible regions. As an analogy, a rigidity analysis would characterize the wiggling of fingers on a single hand as partly correlated, whereas the finger motions from two separate hands are uncorrelated. However, if the hands are clasped, the mobility of all fingers is greatly diminished due to being packed in an interlaced fashion. Thus, the CC-plot highlights channels of communications that are intrinsic to the *skeletal structure* of the protein, but the amplitude of motions that run through these channels are not quantified. In other words, thermodynamics and mechanics is quantified, not kinetics.

The CC plots for all six scFv structures are provided in **Figure S10**. In all cases, the VH domain is primarily composed of one large rigid cluster, punctuated by several flexible loops. There is more variability within the VL domains, which ranges from being mostly rigid in the wild type and VH P101D mutants to intrinsically flexible in the triple mutant. Moreover, the VH and VL domains are flexibly coupled to one other. The CDRs within each domain can be highly correlated as well, especially the H1/H2 and L1/L3 pairs.

**Figure 8:**
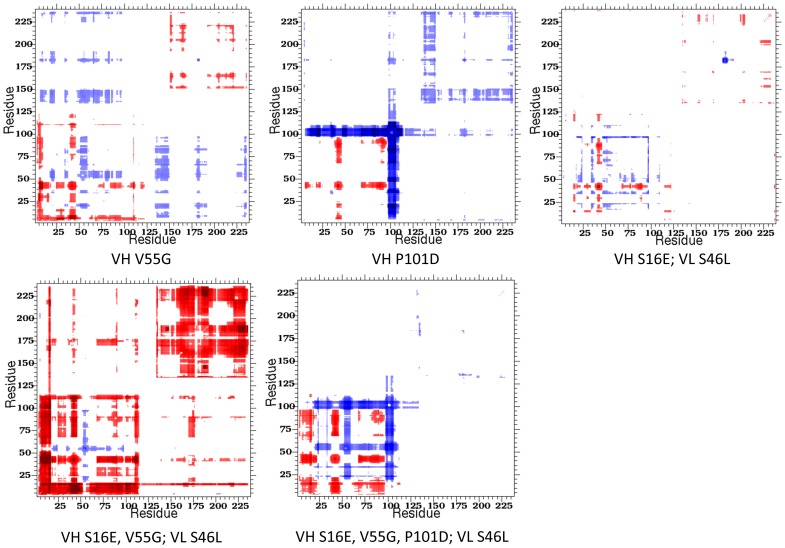
Cooperativity correlation difference plots highlight differences in pairwise mechanical couplings between the wild type and each mutant. Red indicates increased correlated flexibility within the mutant structure, whereas blue indicates increased correlated rigidity. White indicates no change. Notice in most mutants (i.e., triple mutant), changes in cooperativity correlation occur throughout the Fv structure, whereas they are primarily isolated to the VH domain in the quadruple mutant.

Changes within CC highlight the sensitivity of rigidity properties to mutation, which is consistent with a number of our prior works [4], [25], [33], [34], [35]. **Figure 8** plots ΔCC values, using Z-scores per pixel, for each of the mutant structures. Blue coloring indicates residue pairs that are more likely to be rigidly correlated, whereas red indicates residue pairs more likely to be flexibly correlated. These results highlight the connection between backbone flexibility and pairwise couplings. Most of the increases in backbone rigidity occur at locations where increased rigidly correlations also occur. For example, the frequently rigidified CDR H2 shows increased rigidly correlations in all cases where the constituent VH V55G mutation occurs. The connection is even stronger for CDR H3 and its VH P101D mutation. Although more complicated, residues with increased flexibility show an analogous effect. For example, correlated flexibility increases are observed in all of the β4/β5 loops.

### Effects of Rigidity and Stability Changes on Antigen Affinity

The five mutant scFv fragments are composed of various combinations of four constituent mutations, two of which (VH V55G and VH P101D) are located in CDRs. These two mutations lead to significant rigidity increases, yet antigen-binding experiments indicate that all mutants considered retain full antigen-binding affinity [18], [19]. Interestingly, this indicates that the loss of the flexibility in CDR H2 and H3 is not critical for the antigen binding affinity. This observation is consistent with antibody evolution experiments that show, from germline to affinity-mature, somatic mutations constrain conformational heterogeneity to preorder the antigen-binding site to favor association [65], [66]. Thus, there is a degree of plasticity within the constraints imposed on antibody rigidity vis-à-vis antigen binding, including within CDRs. However, it is also worth noting that the flexibility changes observed herein are milder than those that occur during the affinity maturation process (unpublished results).

### Enthalpy-Entropy Compensation upon Mutation

The molecular structure in the local neighborhood of a mutation will accommodate the new residue while respecting local geometrical constraints and network constraints imposed by the protein. These structural constraints are reflected in the native state ensemble of constraint topologies, where only certain modifications to the constraint topologies are thermodynamically accessible. For example, for local optimization of interactions to take place, flexibility may need to be created that was not present in the wild type protein by breaking H-bonds remotely from the mutation. From a thermodynamic point of view, the nature of the most probable constraint topologies will shift through enthalpy-entropy compensation.

It is common to imagine that stabilizing mutations will *decrease* both enthalpy and entropy because rigidity will increase through favorable enthalpic interactions, which causes a decrease in conformational entropy. However, tied to this process, we find that flexibility often increases with a commensurate weakening of the HBN far from the mutation, thus providing a counteracting enthalpy-entropy compensation mechanism where both enthalpy and conformational entropy *increase*. Similar observations have been made by NMR spectroscopy [67]. The molecular details that involve rearrangement of H-bonds and other constraint types may be complicated, but it is pleasing to view this counteracting effect in terms of Le Châtelier’s principle applied to the native state ensemble, where the equilibrium adjusts in a way to counteract the perturbation.

Actually, the change in protein stability must be determined by comparing differences is free energy changes due to mutation between the native and denatured states [68]. However, the native state changes discussed here remain valid because this equilibrium shift is confined to be within an ensemble of conformations comprising the native state. Moreover, Le Châtelier’s principle will equally apply to destabilizing mutants, which is demonstrated simply by looking at the mutations in the reverse direction. Starting with one of the mutants in the dataset considered herein, and mutating it back to the wild type, reveals that local flexibility increase with less favorable enthalpic interactions will cause rigidification far from the mutant. In this context, Le Châtelier’s principle indicates that counteracting changes in rigidity and flexibility will occur at remote sites to globally restore the balance between rigidity and flexibility within protein structures.

**Figure 9:**
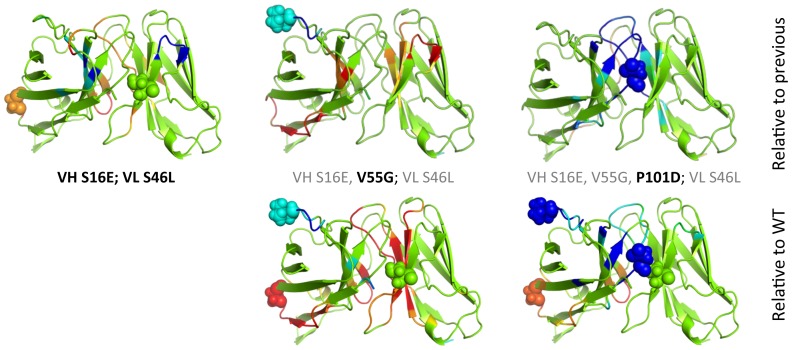
Using the same coloring scheme as Figure 5, rigidity/flexibility changes that occur as additional mutations are added are described. The top row compares, from left to right, the double mutant to the wild type, the triple mutant to the double, and the quadruple to the double, which corresponds to the fewest number of per residue changes. The bottom row re-plots the corresponding structures from **Figure 5**, which are relative to wild type, so the per residue effects can be compared to the global changes.

It is worth stressing that the five mutants considered herein are not independent. That is, the triple mutant is composed of the two residue changes within the double mutant plus one more (VH V55G). Similarly, the quadruple mutant is composed of the three changes within the triple mutant plus one more (VH P101D). Both of these “plus one more” residue changes correspond to the two characterized single mutants. As such, this dataset provides an interesting opportunity to characterize how much the rigidity/flexibility properties change as subsequent mutations are introduced. Using the same coloring scheme as **Figure 5**, **Figure 9** compares the changes in rigidity/flexibility that occur on each additional mutant to the changes within that structure relative to wild-type, which reveals some interesting observations. There is a mix of both strengthening and reversing the changes in the double with respect to wild type when the VH V55G mutation is added to form the triple mutant. For example, the flexibility within VH S16E is increased, whereas the increased rigidity in the β-hairpin near VL S46L becomes flexible relative to wild type. The increase in flexibility that occurs in the double mutant VH β4/β5 loop is maintained in both the triple and quadruple mutants. The increased rigidity at VH V55G that occurs in the triple mutant is similarly maintained in the quadruple, although it is intensified.

The size, scale and frequency of these effects are similar to what is observed in the two single mutants. Across this mutational scheme, the changes in CC are consistent with the presented changes in FI (results not shown). Taken together, these results further underscore how sensitive dynamical properties are to minimal perturbations, which is the chief conclusion of one of our recent reports [58]. Moreover, they demonstrate that in some instances the effects of individual mutations are largely independent from each other, whereas in other instances a single mutation can completely reverse the change at a distal position.

## Conclusions

Predicting how proteins will respond to mutation remains an important open problem. Stabilizing mutations may rigidify local regions through optimized enthalpic interactions. On the other hand, over rigidification can be entropically destabilized [15]. To better understand the relationships between structure and thermodynamics, we characterize the flexibility changes that occur over a set of stabilizing mutants in the scFv fragment of the anti-LTβR antibody. This dataset is ideal because the number of mutations is relatively small, stability gains are significant, and all five mutants structures conserve function by retaining antigen-binding affinity.

Backbone flexibility is qualitatively conserved across the mutant antibodies, but there are statistically significant changes 24% of the time. Interestingly, increases in flexibility are more common than increases in rigidity, and these changes tend to occur at greater distances from the mutation(s). Individual mutants exhibit tendencies that are significantly skewed towards increased rigidity or flexibility. For example, increased flexibility is most common in the VH V55G single mutant and the triple mutant, whereas increased rigidity is predominant in the VH P101D single mutant and the quadruple mutant. Increased rigidity and flexibility are more balanced in the double and quadruple mutants. Furthermore, consistent with our prior works [4], [25], [33], [34], [35], the way flexibility propagates through the protein network via mechanical couplings is more variable due to the sensitivity within allosteric mechanisms [51], [69]. An important component underlying these observations is the role the HBN plays. We found the HBN largely controls molecular mechanisms responsible for the redistribution of flexibility upon site directed mutation, albeit in complex and unexpected ways due to the nonadditive and long-range nature of network rigidity.

We examined the HBNs for each mutant and wild type to identify specific molecular origins of the mechanical response. In the three antibodies where the VH V55G mutation is observed, a local rigidification occurs within CDR H2 based on the formation of new H-bonds relative to wild type. Similarly, new H-bonds within CDR H3 occur in the two instances of the VH P101D mutation. Against the naïve expectation, loss of the proline actually locally rigidifies the structure through optimization of the HBN. All of these mutations also lead to long-range effects related to weakening of the HBN as H-bonds are lost elsewhere, corresponding to increased flexibility. In particular, this is most prominent in the VH β2/β3 and β4/β5 loops on the polar opposite side of the scFv structure. While differences in the HBN between a mutant and wild type provides mechanistic insight behind the changes that occur in rigidity and flexibility, there is no direct correlation between any of the three quantities: H-bond density, FI (rigidity/flexibility) or RMSF (dynamics). The lack of direct correlations between backbone metrics characterizing structure, mechanical response and dynamics underscores that their interrelationships are (at least in part) intrinsically related to subtle long-range collective properties, which can be sensitive to even single mutations.

In summary, the redistribution of flexibility in stabilizing mutations within the scFv fragment of the anti-LTβR antibody will generally involve long-range effects that do not trace to a single structural perturbation, but rather enthalpy-entropy compensation linked to rigidity ⇔ flexibility equilibrium shifts. Although the molecular mechanisms underlying these shifts are largely controlled by alterations within the H-bond network, the link between structure, network rigidity and dynamics remains nebulous, presumably due to the nonadditive and long-range nature of network rigidity. Nevertheless, Le Châtelier’s principle can be applied as a rule of thumb to make credible predictions of long-range effects on protein flexibility upon mutation that might otherwise seem counterintuitive.

## Supporting Information

Figure S1 **Root mean square deviations (Cα) for the VH S16E; VL S46L double mutant molecular dynamics trajectory.** Shown are the global RMSD for the full scFv structure and the two constituent domains considered independently. The small fluctuations within the domains highlight that the global fluctuations are caused by frustration along the domain interface, where the two domains are continually rearranging relative to each other.
(TIF)

Figure S2 **Cluster size of the ten representative structures sampled from the MD simulations.** The values provided in the legend are the total number of frames represented by the top ten clusters.
(TIF)

Figure S3 **The DCM is parameterized by fitting to experimental heat capacity curves.** The best-fit curve for the VH P101D mutant is shown as a black solid line, whereas black circles correspond to the experimental values.
(TIF)

Figure S4 **Scatter plot of the cluster-weighted average** ***T_m_*** **values compared to the experimental values.** The Pearson correlation is 0.72 and the regression *R^2^* is 0.51.
(TIF)

Figure S5 **Computational predictions of** ***T_m_*** **values.** (A) The predicted (red) and experimental (black) *T_m_* values are compared. The unfilled circles correspond to the ten representative structures, whereas the solid red circles correspond to the cluster-weighted averages. As discussed in the text, the error in the wild type prediction is greatest, which corresponds to the only case where the experimental value does not fall within the representative structure range. The percent error for each representative structure and the cluster-weighted averages are presented in panel (B).
(TIF)

Figure S6 **Differences in backbone flexibility are indicated by z-scores using Eq. (3) from above.** Positive values correspond to increased flexibility within the mutant, whereas negative values correspond to increased rigidity. Values within the range of ±2.33 are considered to have no change; values of ±(2.33–3.33) are considered to have moderate changes; and values beyond ±3.33 define large changes. The z-score representation of differences in backbone flexibility quantifies the significance of the observed changes that include both local and non-local changes in rigidity or flexibility.
(TIF)

Figure S7 **The H-bond networks for the wild type and mutant structures are indicated.** White nodes denote H-bond donor and acceptor atoms, and colored edges represent H-bond occupancy across the molecular dynamics simulation trajectory. Black corresponds to H-bonds present greater than 90% of the simulation; blue corresponds to 70–90%; and green corresponds to 50–70%. Because we are primarily interested in stronger H-bonds, those present less than 50% of the time are not shown. The red circle highlights the interfacial region around proline 104 where there are significant changes in the H-bond network within the two structures that include the P104D mutation.
(TIF)

Figure S8 **Sequence of the wild type anti-LTβR single chain Fv fragment with key features indicated.** Mutant positions are highlighted in red. Complementarity determining regions (CDRs) are highlighted in yellow, the two VH loops with increased flexibility are highlighted in green, and the single chain linker is highlighted in cyan. Residue numbering is based on the Kabat scheme.
(TIF)

Figure S9 **Regions that exhibit large increases in flexibility within the quadruple mutant are identified.** The effects of the quadruple mutation (VH S16E, V56G, P104D; VL S46L) on protein flexibility are displayed the upper right panel. The hydrogen bond network (HBN) within the wild type antibody is displayed in the lower left panel. The two red circles emphasize two regions with significant decreases in the mutant HBN compared to wild type, corresponding to increased flexibility. That is, the loss of the hydrogen bonds highlighted in yellow dashed lines cause the corresponding regions to become more flexible.
(TIF)

Figure S10 **Cooperativity correlation plots reveal intramolecular couplings within structure.** That is, blue corresponds to residue pair correlated rigidity, whereas red correspond to correlated flexibility. White indicates no mechanical coupling between a pair of residues irrespective if the residues are flexible or rigid. For each case, the presented values represent the appropriate weighted average values over each set of ten representative structures sampled from the molecular dynamics trajectory.
(TIF)

Table S1 **Frequency of increased rigidity vs. increased flexibility.** Across the dataset the overall number of residues with increased rigidity (42%) is similar to increased flexibility (58%).
(DOCX)

## References

[1] Fleming PJ, Rose GD (2005) Do all backbone polar groups in proteins form hydrogen bonds? Protein Sci 14: 1911–1917. 15937286 10.1110/ps.051454805 PMC2253345

[2] Livesay DR (2010) Protein dynamics: dancing on an ever-changing free energy stage. Curr Opin Pharmacol 10: 706–708. 20951643 10.1016/j.coph.2010.09.015 PMC2981666

[3] Tokuriki N, Tawfik DS (2009) Stability effects of mutations and protein evolvability. Curr Opin Struct Biol 19: 596–604. 19765975 10.1016/j.sbi.2009.08.003

[4] Verma D, Jacobs DJ, Livesay DR (2012) Changes in lysozyme flexibility upon mutation are frequent, large and long-ranged. PLoS Comput Biol 8: e1002409. 22396637 10.1371/journal.pcbi.1002409 PMC3291535

[5] Yutani K, Ogasahara K, Sugino Y (1985) Effect of amino acid substitutions on conformational stability of a protein. Adv Biophys 20: 13–29. 3914832 10.1016/0065-227x(85)90028-0

[6] Studer RA, Dessailly BH, Orengo CA (2013) Residue mutations and their impact on protein structure and function: detecting beneficial and pathogenic changes. Biochem J 449: 581–594. 23301657 10.1042/BJ20121221

[7] Rader AJ (2009) Thermostability in rubredoxin and its relationship to mechanical rigidity. Phys Biol 7: 16002. 20009190 10.1088/1478-3975/7/1/016002

[8] Radestock S, Gohlke H (2011) Protein rigidity and thermophilic adaptation. Proteins 79: 1089–1108. 21246632 10.1002/prot.22946

[9] van den Burg B, Eijsink VG (2002) Selection of mutations for increased protein stability. Curr Opin Biotechnol 13: 333–337. 12323355 10.1016/s0958-1669(02)00325-7

[10] Lee HJ, Yoon YJ, Jang do S, Kim C, Cha HJ, et al (2008) 15N NMR relaxation studies of Y14F mutant of ketosteroid isomerase: the influence of mutation on backbone mobility. J Biochem 144: 159–166. 18424811 10.1093/jb/mvn053

[11] Liu J, Song J (2009) Insights into protein aggregation by NMR characterization of insoluble SH3 mutants solubilized in salt-free water. PLoS One 4: e7805. 19956763 10.1371/journal.pone.0007805 PMC2776303

[12] Mulder FA, Hon B, Muhandiram DR, Dahlquist FW, Kay LE (2000) Flexibility and ligand exchange in a buried cavity mutant of T4 lysozyme studied by multinuclear NMR. Biochemistry 39: 12614–12622. 11027141 10.1021/bi001351t

[13] Wen Y, Li J, Xiong M, Peng Y, Yao W, et al (2010) Solution structure and dynamics of the I214V mutant of the rabbit prion protein. PLoS One 5: e13273. 20949107 10.1371/journal.pone.0013273 PMC2951349

[14] Yuan X, Werner JM, Lack J, Knott V, Handford PA, et al (2002) Effects of the N2144S mutation on backbone dynamics of a TB-cbEGF domain pair from human fibrillin-1. J Mol Biol 316: 113–125. 11829507 10.1006/jmbi.2001.5329

[15] Hu H, Clarkson MW, Hermans J, Lee AL (2003) Increased rigidity of eglin c at acidic pH: evidence from NMR spin relaxation and MD simulations. Biochemistry 42: 13856–13868. 14636053 10.1021/bi035015z

[16] Verma D, Jacobs DJ, Livesay DR (2010) Predicting the melting point of human C-type lysozyme mutants. Curr Protein Pept Sci 11: 562–572. 20887260 10.2174/138920310794109210 PMC4667962

[17] Jacobs DJ (2012) An interfacial model for protein stability. In: Misra AN, editor. Biophysics: Intech. 91–132.

[18] Jordan JL, Arndt JW, Hanf K, Li G, Hall J, et al (2009) Structural understanding of stabilization patterns in engineered bispecific Ig-like antibody molecules. Proteins 77: 832–841. 19626705 10.1002/prot.22502

[19] Miller BR, Demarest SJ, Lugovskoy A, Huang F, Wu X, et al (2010) Stability engineering of scFvs for the development of bispecific and multivalent antibodies. Protein Eng Des Sel 23: 549–557. 20457695 10.1093/protein/gzq028

[20] Dunitz JD (1995) Win some, lose some: enthalpy-entropy compensation in weak intermolecular interactions. Chem Biol 2: 709–712. 9383477 10.1016/1074-5521(95)90097-7

[21] Vorov OK, Livesay DR, Jacobs DJ (2009) Helix/coil nucleation: a local response to global demands. Biophys J 97: 3000–3009. 19948130 10.1016/j.bpj.2009.09.013 PMC2784565

[22] Vorov OK, Livesay DR, Jacobs DJ (2011) Nonadditivity in conformational entropy upon molecular rigidification reveals a universal mechanism affecting folding cooperativity. Biophys J 100: 1129–1138. 21320459 10.1016/j.bpj.2011.01.027 PMC3037720

[23] Frankel AD (1992) The importance of being flexible. Proc Natl Acad Sci U S A 89: 11653. 1361230 10.1073/pnas.89.24.11653 PMC50613

[24] Hollien J, Marqusee S (1999) A thermodynamic comparison of mesophilic and thermophilic ribonucleases H. Biochemistry. 38: 3831–3836. 10.1021/bi982684h 10090773

[25] Livesay DR, Jacobs DJ (2006) Conserved quantitative stability/flexibility relationships (QSFR) in an orthologous RNase H pair. Proteins 62: 130–143. 16287093 10.1002/prot.20745 PMC4678005

[26] Jacobs DJ, Rader AJ, Kuhn LA, Thorpe MF (2001) Protein flexibility predictions using graph theory. Proteins 44: 150–165. 11391777 10.1002/prot.1081

[27] Jacobs DJ, Thorpe MF (1995) Generic rigidity percolation: The pebble game. Phys Rev Lett 75: 4051–4054. 10059802 10.1103/PhysRevLett.75.4051

[28] Istomin AY, Gromiha MM, Vorov OK, Jacobs DJ, Livesay DR (2008) New insight into long-range nonadditivity within protein double-mutant cycles. Proteins 70: 915–924. 17803237 10.1002/prot.21620 PMC4667956

[29] Jacobs DJ, Dallakyan S (2005) Elucidating protein thermodynamics from the three-dimensional structure of the native state using network rigidity. Biophys J 88: 903–915. 15542549 10.1529/biophysj.104.048496 PMC1305163

[30] Livesay DR, Dallakyan S, Wood GG, Jacobs DJ (2004) A flexible approach for understanding protein stability. FEBS Lett 576: 468–476. 15498582 10.1016/j.febslet.2004.09.057

[31] Loladze VV, Ermolenko DN, Makhatadze GI (2002) Thermodynamic consequences of burial of polar and non-polar amino acid residues in the protein interior. Journal Of Molecular Biology 320: 343–357. 12079391 10.1016/S0022-2836(02)00465-5

[32] Jacobs DJ, Livesay DR, Hules J, Tasayco ML (2006) Elucidating quantitative stability/flexibility relationships within thioredoxin and its fragments using a distance constraint model. J Mol Biol 358: 882–904. 16542678 10.1016/j.jmb.2006.02.015 PMC4667950

[33] Livesay DR, Huynh DH, Dallakyan S, Jacobs DJ (2008) Hydrogen bond networks determine emergent mechanical and thermodynamic properties across a protein family. Chem Cent J 2: 17. 18700034 10.1186/1752-153X-2-17 PMC2533333

[34] Mottonen JM, Jacobs DJ, Livesay DR (2010) Allosteric response is both conserved and variable across three CheY orthologs. Biophys J 99: 2245–2254. 20923659 10.1016/j.bpj.2010.07.043 PMC3042579

[35] Mottonen JM, Xu M, Jacobs DJ, Livesay DR (2009) Unifying mechanical and thermodynamic descriptions across the thioredoxin protein family. Proteins 75: 610–627. 19004018 10.1002/prot.22273 PMC2972311

[36] Hess B, Kutzner C, Van Der Spoel D, Lindahl E (2008) GROMACS 4: Algorithms for highly efficient load-balanced, and scalable molecular simulation. J Chem Theory Comput 4. 10.1021/ct700301q 26620784

[37] Van Der Spoel D, Lindahl E, Hess B, Groenhof G, Mark AE, et al (2005) GROMACS: fast, flexible, and free. J Comput Chem 26: 1701–1718. 16211538 10.1002/jcc.20291

[38] Lindorff-Larsen K, Piana S, Palmo K, Maragakis P, Klepeis JL, et al (2010) Improved side-chain torsion potentials for the Amber ff99SB protein force field. Proteins 78: 1950–1958. 20408171 10.1002/prot.22711 PMC2970904

[39] Jorgensen WL, Chandrasekhar J, Madura JD, Impey RW, Klein ML (1983) Comparison of simple potential functions for simulating liquid water. J Chem Phys 79: 926–935.

[40] Nose S (1984) A Molecular-Dynamics Method for Simulations in the Canonical Ensemble. Molecular Physics 52: 255–268.

[41] Hoover WG (1985) Canonical dynamics: Equilibrium phase-space distributions. Phys Rev A 31: 1695–1697. 10.1103/physreva.31.1695 9895674

[42] Nose S (1985) A molecualr dynamics method for simulations in the canonical ensemble. Molecular Physics 52.

[43] Darden TA, York DM, Pedersen LG (1993) Particle mesh Ewald: an N log (N) method for Ewald sums in large systems. J Chem Phys 98: 10089–10092.

[44] Hess B, Bekker H, Berendsen HJC, Fraaije JGEM (1997) LINCS: A linear constraint solver for molecular simulations. Journal of Computational Chemistry 18: 1463–1472.

[45] Miyamoto S, Kollman PA (1992) Settle - an Analytical Version of the Shake and Rattle Algorithm for Rigid Water Models. Journal of Computational Chemistry 13: 952–962.

[46] Banfield MJ, King DJ, Mountain A, Brady RL (1997) VL:VH domain rotations in engineered antibodies: crystal structures of the Fab fragments from two murine antitumor antibodies and their engineered human constructs. Proteins 29: 161–171. 9329081 10.1002/(sici)1097-0134(199710)29:2<161::aid-prot4>3.0.co;2-g

[47] Nakanishi T, Tsumoto K, Yokota A, Kondo H, Kumagai I (2008) Critical contribution of VH-VL interaction to reshaping of an antibody: the case of humanization of anti-lysozyme antibody, HyHEL-10. Protein Sci 17: 261–270. 18227432 10.1110/ps.073156708 PMC2222731

[48] Narayanan A, Sellers BD, Jacobson MP (2009) Energy-based analysis and prediction of the orientation between light- and heavy-chain antibody variable domains. J Mol Biol 388: 941–953. 19324053 10.1016/j.jmb.2009.03.043

[49] Karpen ME, Tobias DJ, Brooks CL 3rd (1993) Statistical clustering techniques for the analysis of long molecular dynamics trajectories: analysis of 2.2-ns trajectories of YPGDV. Biochemistry 32: 412–420. 8422350 10.1021/bi00053a005

[50] Feig M, Karanicolas J, Brooks CL 3rd (2004) MMTSB Tool Set: enhanced sampling and multiscale modeling methods for applications in structural biology. J Mol Graph Model 22: 377–395. 15099834 10.1016/j.jmgm.2003.12.005

[51] Jacobs DJ, Livesay DR, Mottonen JM, Vorov OK, Istomin AY, et al (2012) Ensemble properties of network rigidity reveal allosteric mechanisms. Methods Mol Biol 796: 279–304. 22052496 10.1007/978-1-61779-334-9_15 PMC4676805

[52] Arnold K, Bordoli L, Kopp J, Schwede T (2006) The SWISS-MODEL workspace: a web-based environment for protein structure homology modelling. Bioinformatics 22: 195–201. 16301204 10.1093/bioinformatics/bti770

[53] Krivov GG, Shapovalov MV, Dunbrack RL Jr (2009) Improved prediction of protein side-chain conformations with SCWRL4. Proteins 77: 778–795. 19603484 10.1002/prot.22488 PMC2885146

[54] Gordon JC, Myers JB, Folta T, Shoja V, Heath LS, et al (2005) H++: a server for estimating pKas and adding missing hydrogens to macromolecules. Nucleic Acids Res 33: W368–371. 15980491 10.1093/nar/gki464 PMC1160225

[55] Li T, Verma D, Malgorzata BT, Casas-Finet J, Livesay DR, et al. (In press) Thermodynamic and stability characteristics of antibody fragment complexes. Prot Pept Lett. 10.2174/09298665113209990051 PMC4667953 23855672

[56] Johnson G, Wu TT (2001) Kabat Database and its applications: future directions. Nucleic Acids Res 29: 205–206. 11125092 10.1093/nar/29.1.205 PMC29771

[57] Li T, Verma D, Tracka MB, Casas-Finet J, Livesay DR, et al. (2013) Thermodynamic Stability and Flexibility Characteristics of Antibody Fragment Complexes. Protein and peptide letters. 10.2174/09298665113209990051 PMC4667953 23855672

[58] Verma D, Jacobs DJ, Livesay DR (2012) Changes in Lysozyme Flexibility upon Mutation Are Frequent, Large and Long-Ranged. PLoS Comput Biol 8: e1002409. 22396637 10.1371/journal.pcbi.1002409 PMC3291535

[59] Verma D, Jacobs DJ, Livesay DR (2013) Variations within class-A beta-lactamase physiochemical properties reflect evolutionary and environmental patterns, but not antibiotic specificity. PLoS Comput Biol 9: e1003155. 23874193 10.1371/journal.pcbi.1003155 PMC3715408

[60] Pfleger C, Gohlke H (2013) Efficient and robust analysis of biomacromolecular flexibility using ensembles of network topologies based on fuzzy noncovalent constraints. Structure In press. 10.1016/j.str.2013.07.012 23994009

[61] Fields PA (2001) Review: Protein function at thermal extremes: balancing stability and flexibility. Comp Biochem Physiol A Mol Integr Physiol 129: 417–431. 11423314 10.1016/s1095-6433(00)00359-7

[62] Jaenicke R, Zavodszky P (1990) Proteins under extreme physical conditions. FEBS Lett 268: 344–349. 2200715 10.1016/0014-5793(90)81283-t

[63] Kamerzell TJ, Middaugh CR (2008) The complex inter-relationships between protein flexibility and stability. J Pharm Sci 97: 3494–3517. 18186490 10.1002/jps.21269

[64] Nisius L, Grzesiek S (2012) Key stabilizing elements of protein structure identified through pressure and temperature perturbation of its hydrogen bond network. Nat Chem 4: 711–717. 22914191 10.1038/nchem.1396

[65] Manivel V, Sahoo NC, Salunke DM, Rao KV (2000) Maturation of an antibody response is governed by modulations in flexibility of the antigen-combining site. Immunity 13: 611–620. 11114374 10.1016/s1074-7613(00)00061-3

[66] Zimmermann J, Oakman EL, Thorpe IF, Shi X, Abbyad P, et al (2006) Antibody evolution constrains conformational heterogeneity by tailoring protein dynamics. Proc Natl Acad Sci U S A 103: 13722–13727. 16954202 10.1073/pnas.0603282103 PMC1564241

[67] Stone MJ (2001) NMR relaxation studies of the role of conformational entropy in protein stability and ligand binding. Acc Chem Res 34: 379–388. 11352716 10.1021/ar000079c

[68] Fu H, Grimsley G, Scholtz JM, Pace CN (2010) Increasing protein stability: importance of DeltaC(p) and the denatured state. Protein Sci 19: 1044–1052. 20340133 10.1002/pro.381 PMC2868246

[69] Livesay DR, Kreth KE, Fodor AA (2012) A critical evaluation of correlated mutation algorithms and coevolution within allosteric mechanisms. Methods Mol Biol 796: 385–398. 22052502 10.1007/978-1-61779-334-9_21

