# Supplementary figures and images for: Redistribution of Flexibility in Stabilizing Antibody Fragment Mutants Follows Le Châtelier’s Principle
Source: PLoS One. 2014 Mar 26;9(3):e92870. doi: 10.1371/journal.pone.0092870 (PMC3966838; doi:10.1371/journal.pone.0092870)

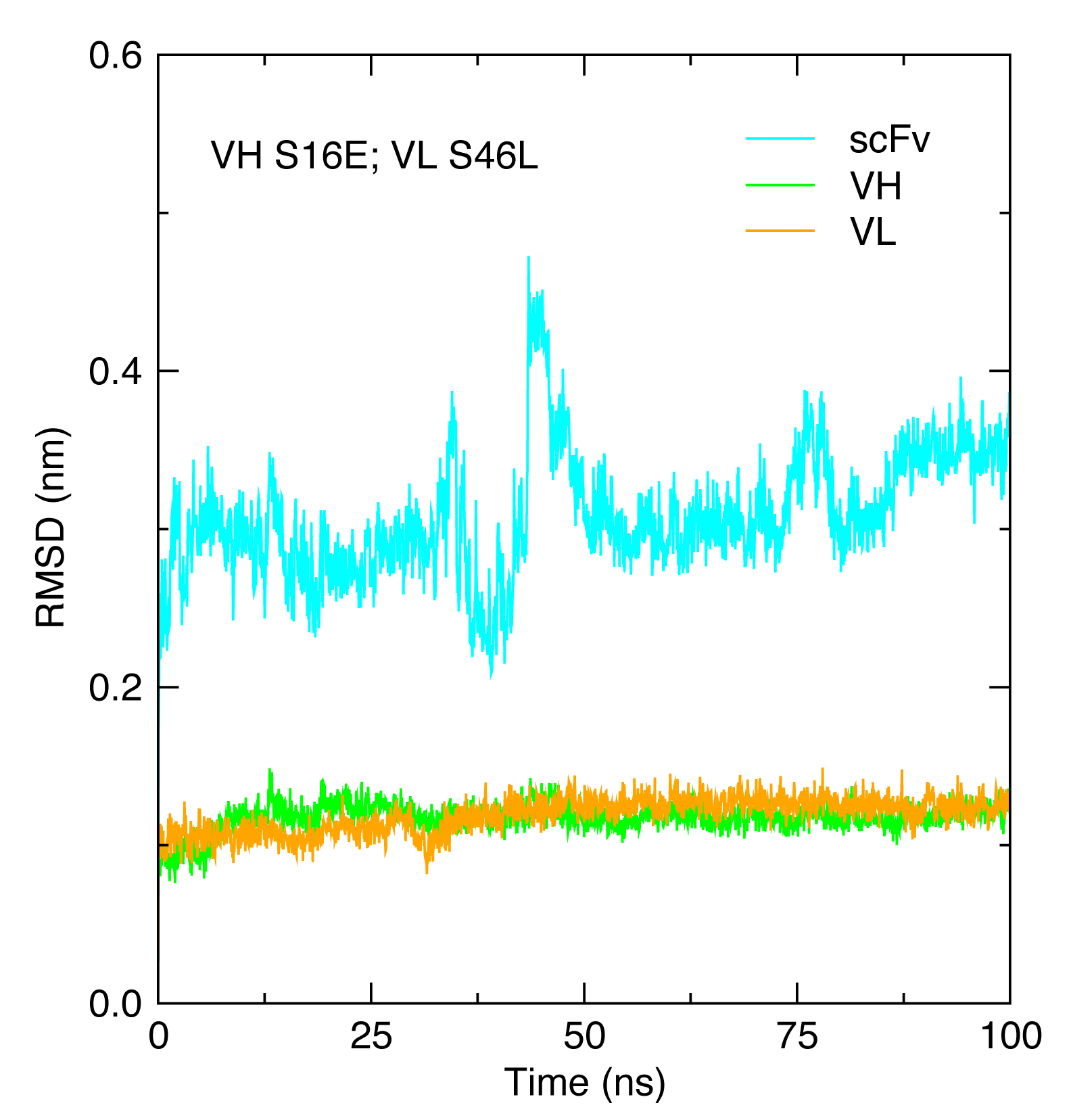

Supplement: Figure S1 — Root mean square deviations (Cα) for the VH S16E; VL S46L double mutant molecular dynamics trajectory. Shown are the global RMSD for the full scFv structure and the two constituent domains considered independently. The small fluctuations within the domains highlight that the global fluctuations are caused by frustration along the domain interface, where the two domains are continually rearranging relative to each other. (TIF) [file pone.0092870.s001.tif]

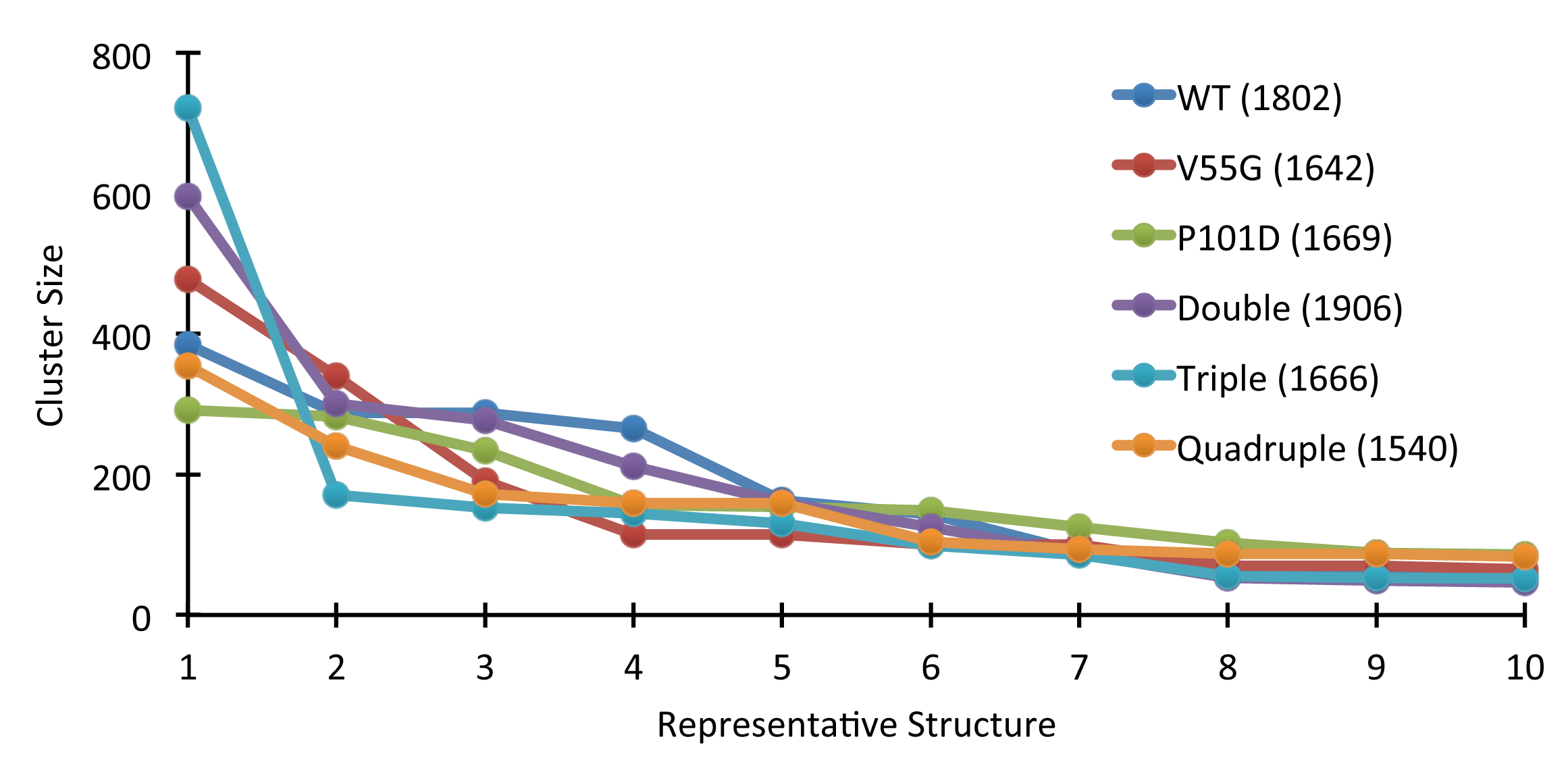

Supplement: Figure S2 — Cluster size of the ten representative structures sampled from the MD simulations. The values provided in the legend are the total number of frames represented by the top ten clusters. (TIF) [file pone.0092870.s002.tif]

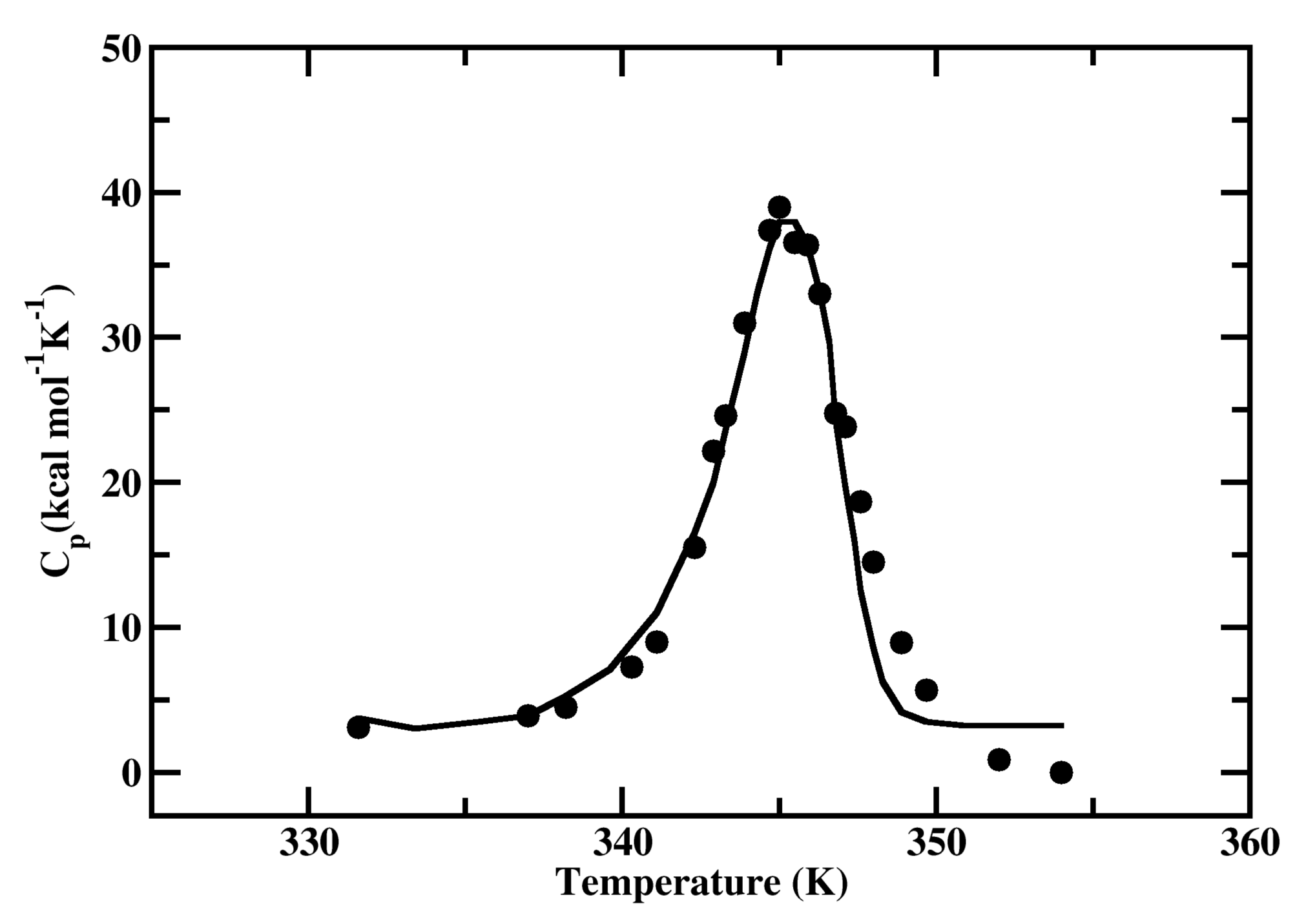

Supplement: Figure S3 — The DCM is parameterized by fitting to experimental heat capacity curves. The best-fit curve for the VH P101D mutant is shown as a black solid line, whereas black circles correspond to the experimental values. (TIF) [file pone.0092870.s003.tif]

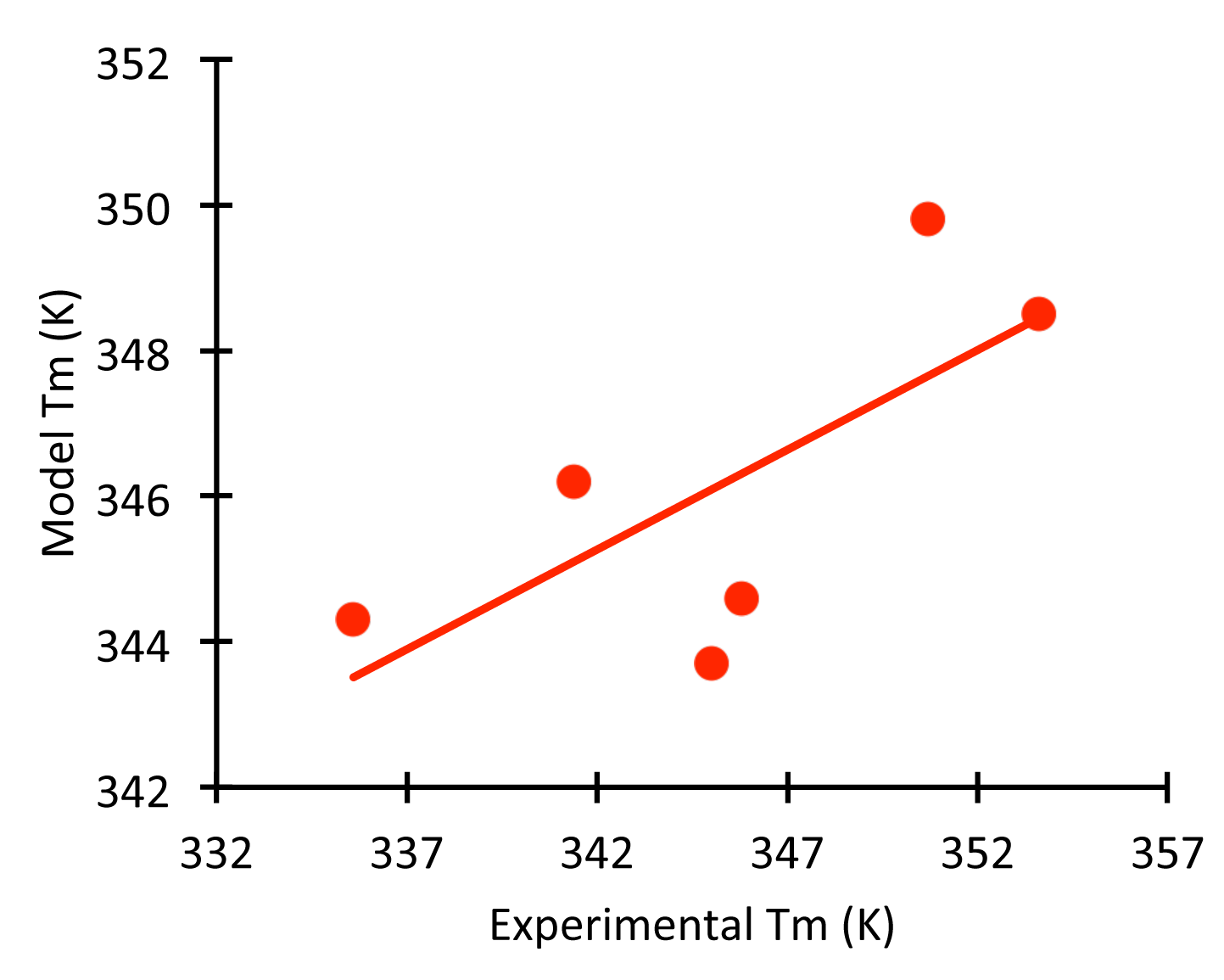

Supplement: Figure S4 — Scatter plot of the cluster-weighted average Tm values compared to the experimental values. The Pearson correlation is 0.72 and the regression R2 is 0.51. (TIF) [file pone.0092870.s004.tif]

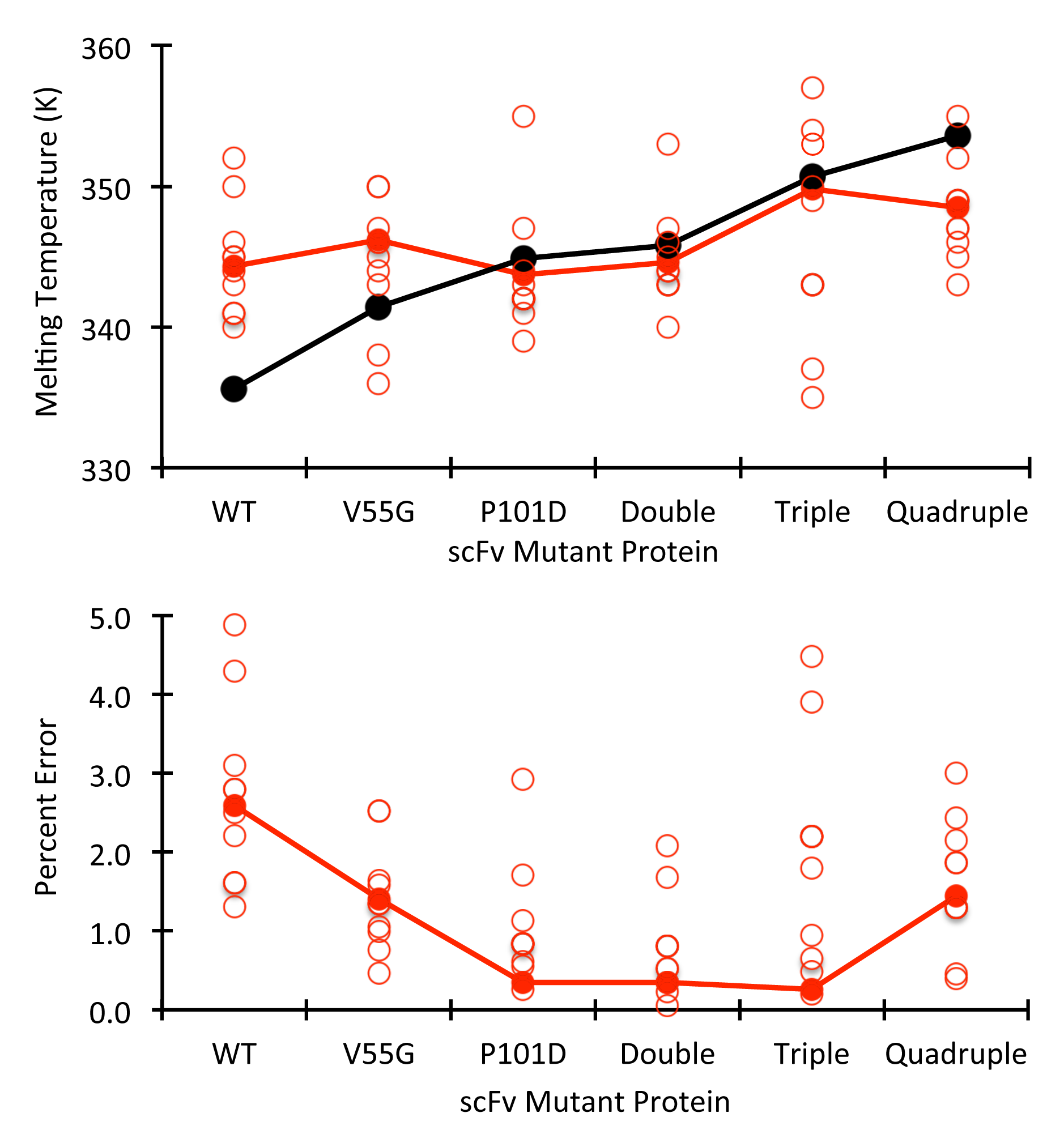

Supplement: Figure S5 — Computational predictions of Tm values. (A) The predicted (red) and experimental (black) Tm values are compared. The unfilled circles correspond to the ten representative structures, whereas the solid red circles correspond to the cluster-weighted averages. As discussed in the text, the error in the wild type prediction is greatest, which corresponds to the only case where the experimental value does not fall within the representative structure range. The percent error for each representative structure and the cluster-weighted averages are presented in panel (B). (TIF) [file pone.0092870.s005.tif]

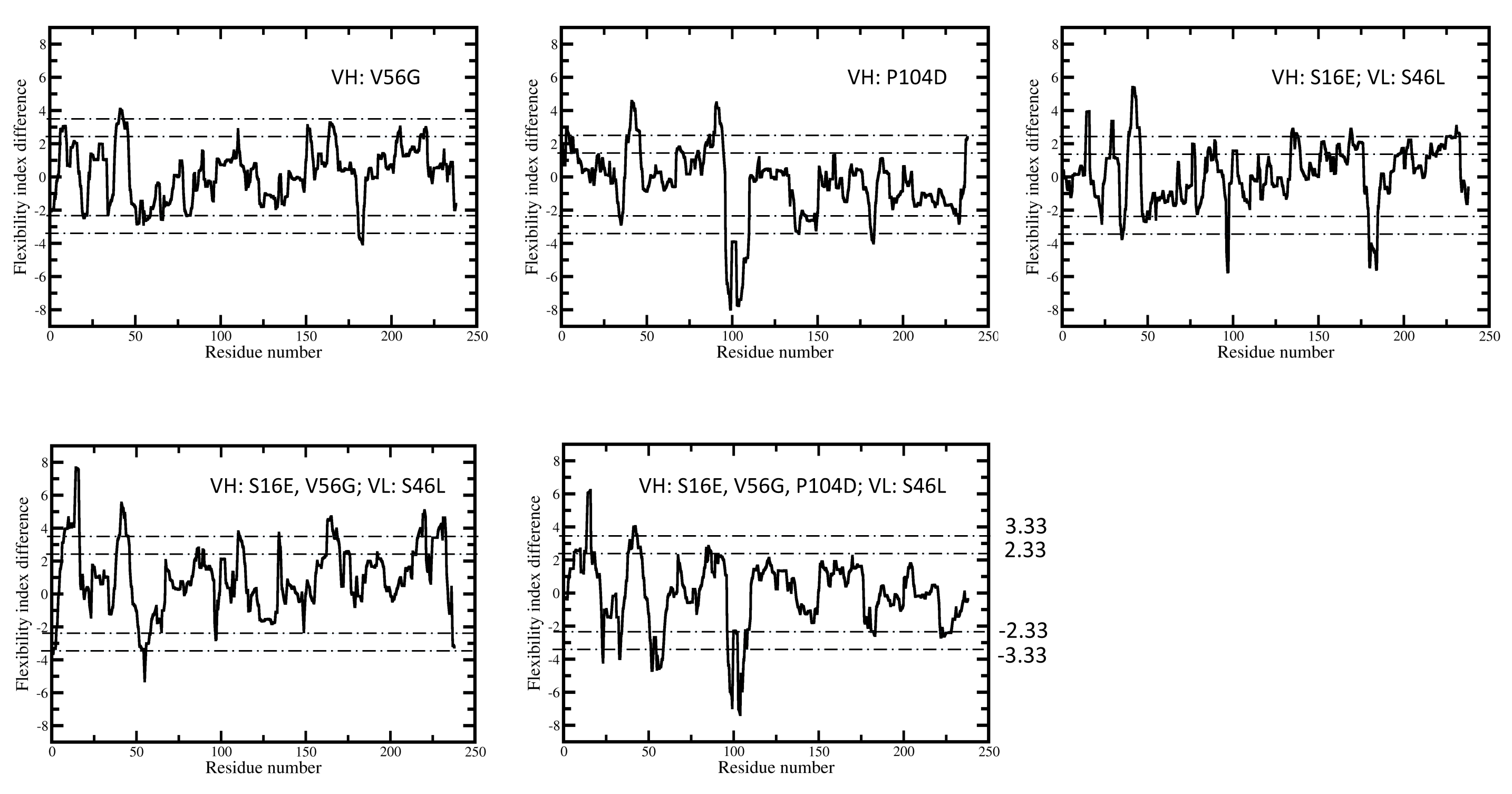

Supplement: Figure S6 — Differences in backbone flexibility are indicated by z-scores using Eq. (3) from above. Positive values correspond to increased flexibility within the mutant, whereas negative values correspond to increased rigidity. Values within the range of ±2.33 are considered to have no change; values of ±(2.33–3.33) are considered to have moderate changes; and values beyond ±3.33 define large changes. The z-score representation of differences in backbone flexibility quantifies the significance of the observed changes that include both local and non-local changes in rigidity or flexibility. (TIF) [file pone.0092870.s006.tif]

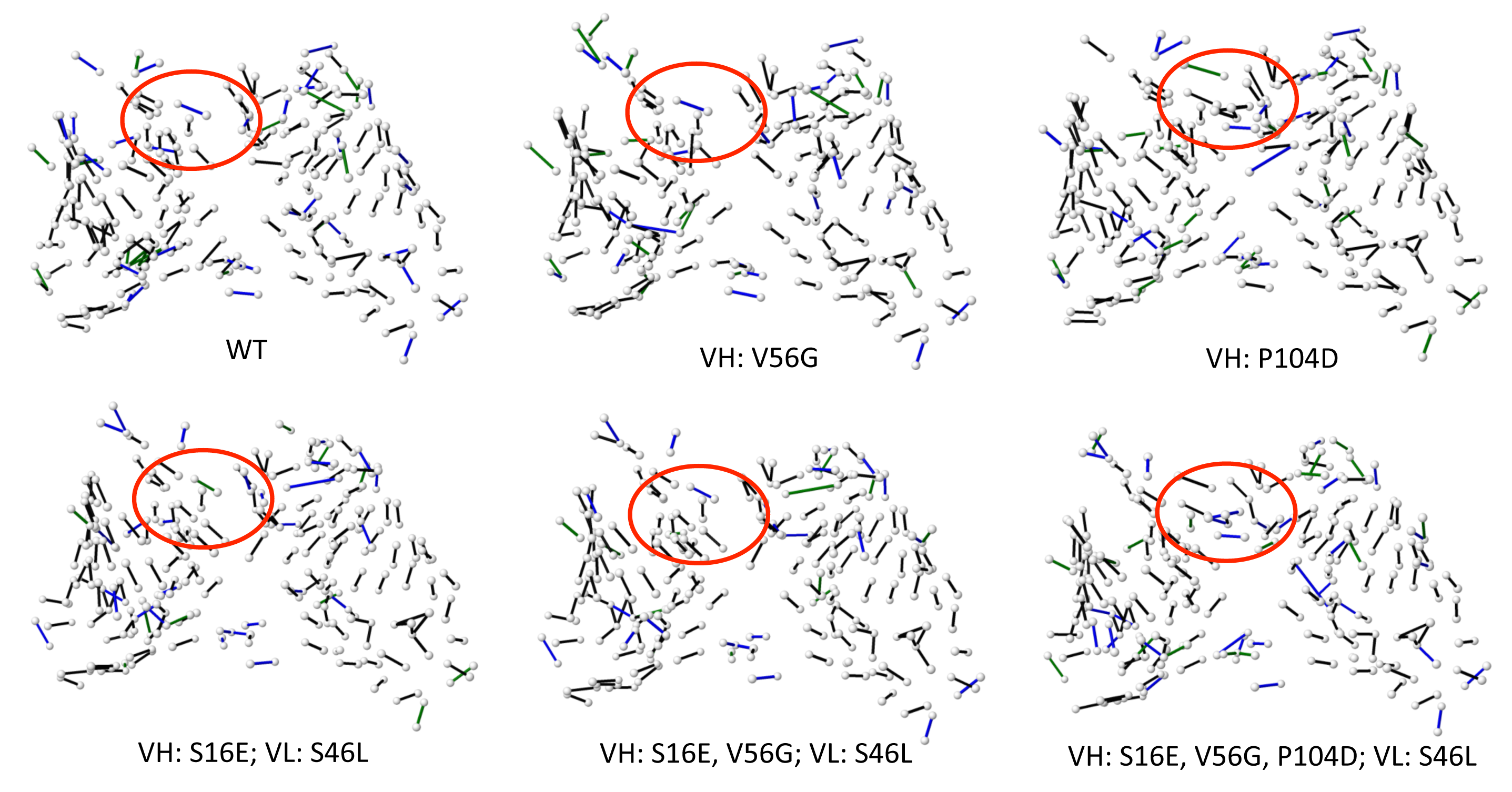

Supplement: Figure S7 — The H-bond networks for the wild type and mutant structures are indicated. White nodes denote H-bond donor and acceptor atoms, and colored edges represent H-bond occupancy across the molecular dynamics simulation trajectory. Black corresponds to H-bonds present greater than 90% of the simulation; blue corresponds to 70–90%; and green corresponds to 50–70%. Because we are primarily interested in stronger H-bonds, those present less than 50% of the time are not shown. The red circle highlights the interfacial region around proline 104 where there are significant changes in the H-bond network within the two structures that include the P104D mutation. (TIF) [file pone.0092870.s007.tif]

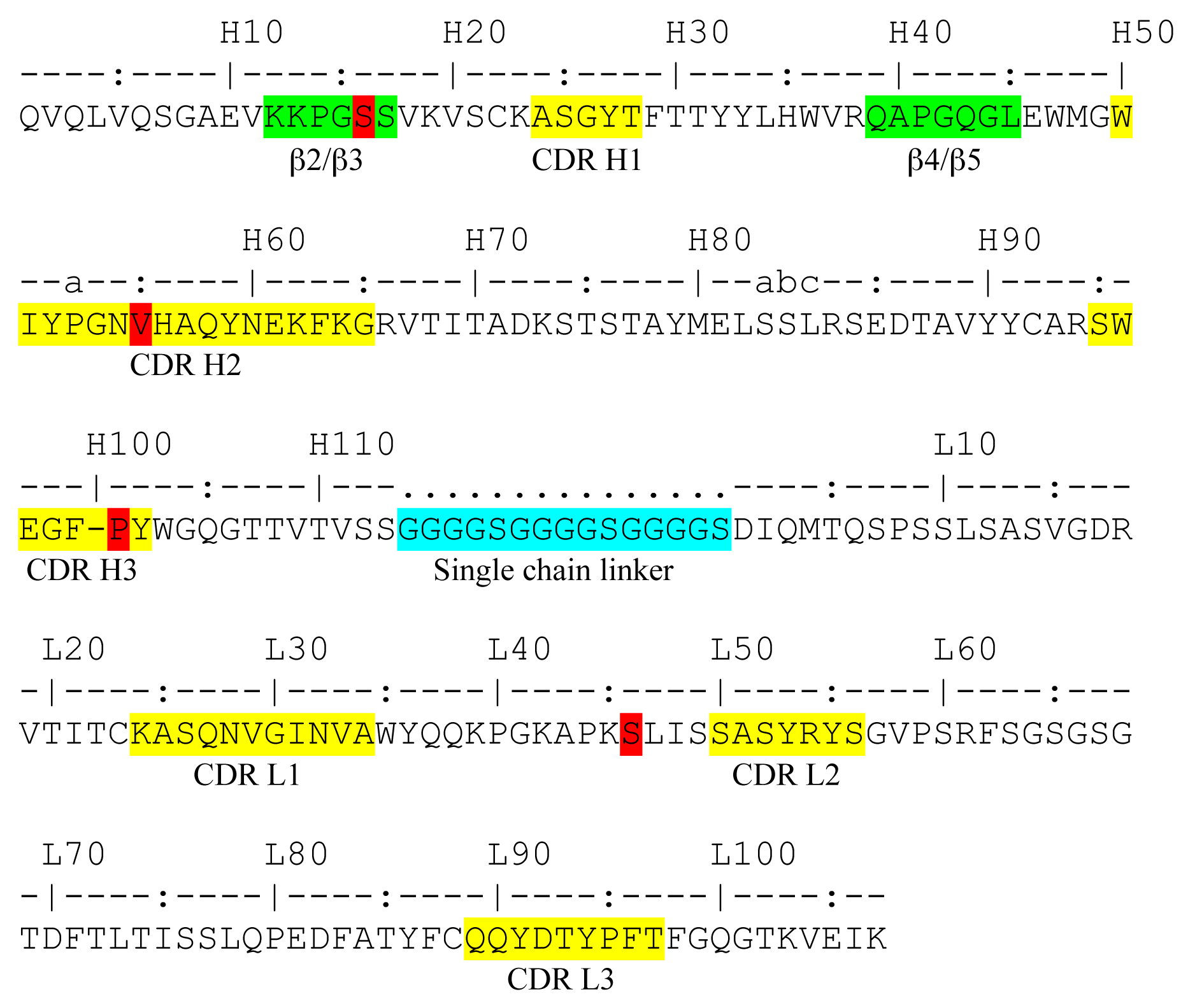

Supplement: Figure S8 — Sequence of the wild type anti-LTβR single chain Fv fragment with key features indicated. Mutant positions are highlighted in red. Complementarity determining regions (CDRs) are highlighted in yellow, the two VH loops with increased flexibility are highlighted in green, and the single chain linker is highlighted in cyan. Residue numbering is based on the Kabat scheme. (TIF) [file pone.0092870.s008.tif]

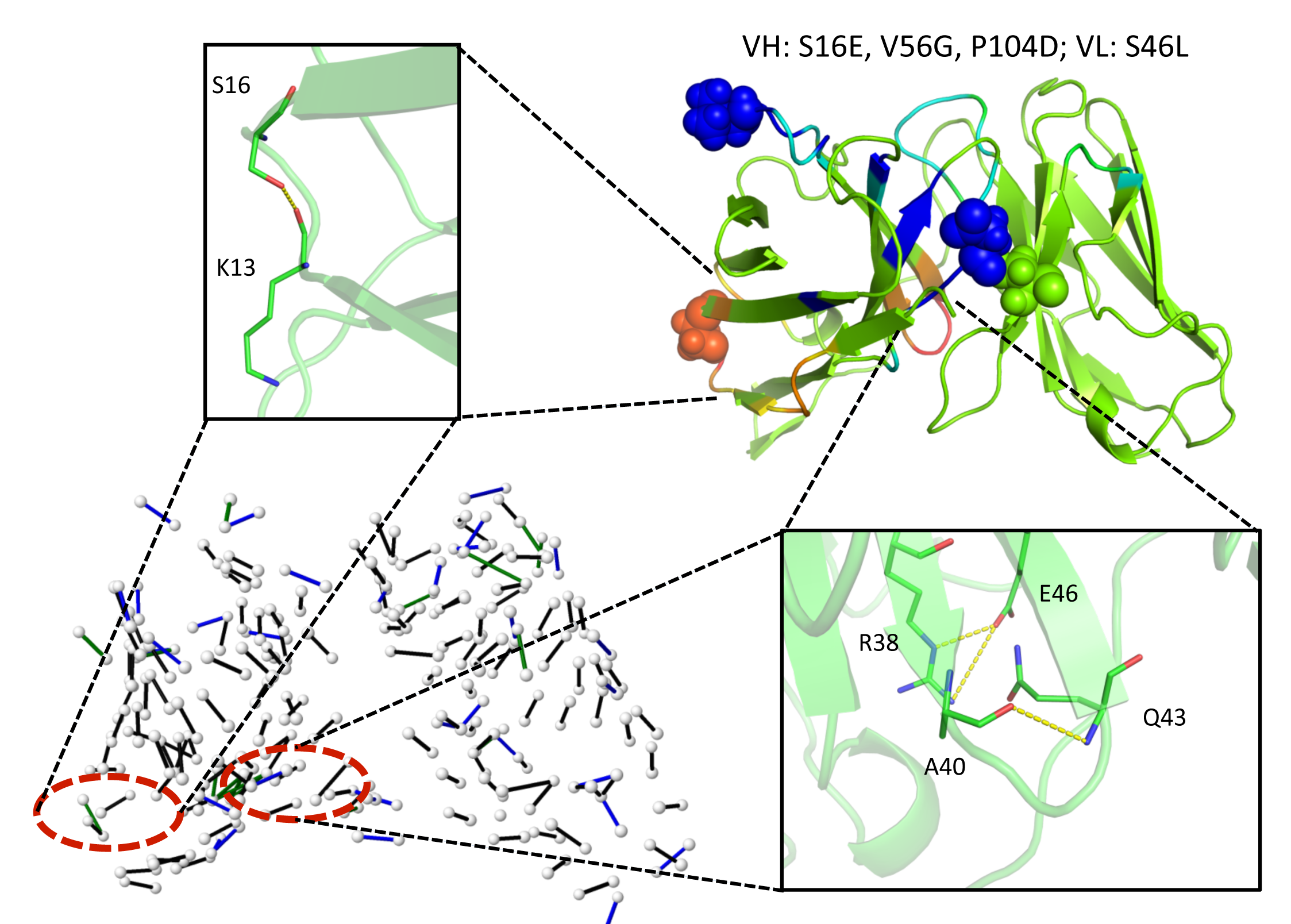

Supplement: Figure S9 — Regions that exhibit large increases in flexibility within the quadruple mutant are identified. The effects of the quadruple mutation (VH S16E, V56G, P104D; VL S46L) on protein flexibility are displayed the upper right panel. The hydrogen bond network (HBN) within the wild type antibody is displayed in the lower left panel. The two red circles emphasize two regions with significant decreases in the mutant HBN compared to wild type, corresponding to increased flexibility. That is, the loss of the hydrogen bonds highlighted in yellow dashed lines cause the corresponding regions to become more flexible. (TIF) [file pone.0092870.s009.tif]

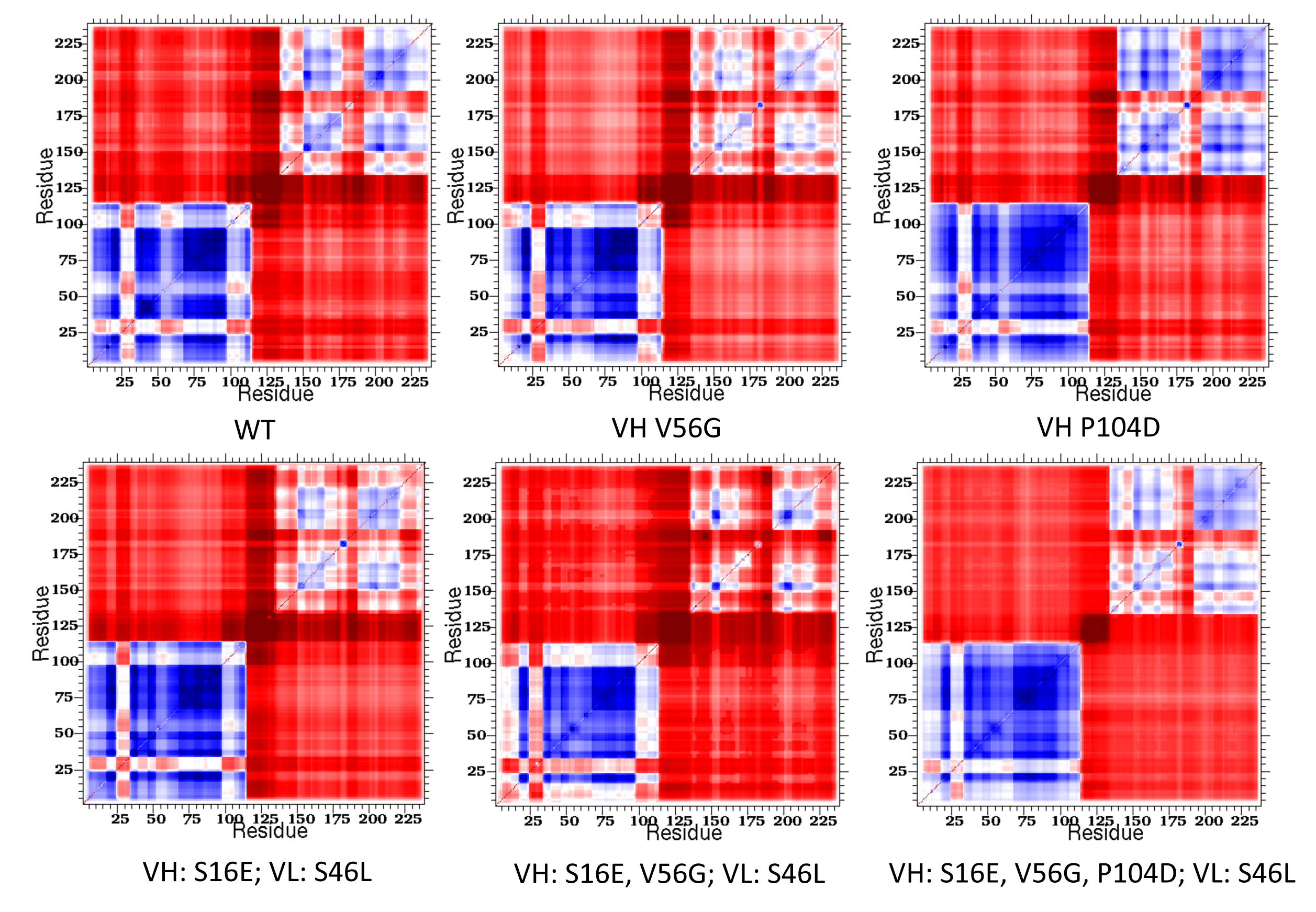

Supplement: Figure S10 — Cooperativity correlation plots reveal intramolecular couplings within structure. That is, blue corresponds to residue pair correlated rigidity, whereas red correspond to correlated flexibility. White indicates no mechanical coupling between a pair of residues irrespective if the residues are flexible or rigid. For each case, the presented values represent the appropriate weighted average values over each set of ten representative structures sampled from the molecular dynamics trajectory. (TIF) [file pone.0092870.s010.tif]
