# Supplementary material for: Redistribution of Flexibility in Stabilizing Antibody Fragment Mutants Follows Le Châtelier’s Principle
Source: PLoS One. 2014 Mar 26;9(3):e92870. doi: 10.1371/journal.pone.0092870 (PMC3966838; doi:10.1371/journal.pone.0092870)
Supplement: Table S1 — Frequency of increased rigidity vs. increased flexibility. Across the dataset the overall number of residues with increased rigidity (42%) is similar to increased flexibility (58%). (DOCX) [file pone.0092870.s011.docx]

**Table S1:** Frequency of increased rigidity vs. increased flexibility.

| Response | Flexibility Increases | | Rigidity Increases | |
| --- | --- | --- | --- | --- |
|  | 2.3 ≤ *x*< 3.3 | 3.3 ≤ *x* | -3.3 < *x*≤ -2.3 | *x ≤*-3.3 |
| *Whole Protein* | | | | |
| VH: V55G | 21 | 3 | 9 | 3 |
| VH: P101D | 9 | 6 | 14 | 16 |
| VH: S16E; VL: S46L | 16 | 6 | 7 | 7 |
| VH: S16E, V55G; VL: S46L | 16 | 39 | 8 | 4 |
| VH: S16E, V55G, P101D; VL: S46L | 12 | 6 | 13 | 14 |
| **Total** | **74** | **60** | **51** | **44** |
| *Complementarity Determining Regions* | | | | |
| VH: V55G | 1 | 0 | 4 | 3 |
| VH: P101D | 0 | 0 | 1 | 8 |
| VH: S16E; VL: S46L | 1 | 1 | 3 | 3 |
| VH: S16E, V55G; VL: S46L | 2 | 4 | 3 | 3 |
| VH: S16E, V55G, P101D; VL: S46L | 0 | 0 | 9 | 7 |
| **Total** | **4** | **5** | **20** | **24** |
| *Non-CDR Loops* | | | | |
| VH: V55G | 5 | 3 | 2 | 0 |
| VH: P101D | 3 | 5 | 6 | 4 |
| VH: S16E; VL: S46L | 5 | 5 | 0 | 0 |
| VH: S16E, V55G; VL: S46L | 5 | 16 | 1 | 1 |
| VH: S16E, V55G, P101D; VL: S46L | 8 | 3 | 1 | 0 |
| **Total** | **26** | **32** | **10** | **5** |
